# Bre1-dependent H2B ubiquitination promotes homologous recombination by stimulating histone eviction at DNA breaks

**DOI:** 10.1093/nar/gky918

**Published:** 2018-10-10

**Authors:** Sihao Zheng, Dan Li, Zhen Lu, Guangxue Liu, Meng Wang, Poyuan Xing, Min Wang, Yang Dong, Xuejie Wang, Jingyao Li, Simin Zhang, Haoyang Peng, Grzegorz Ira, Guohong Li, Xuefeng Chen

**Affiliations:** 1 Hubei Key Laboratory of Cell Homeostasis, the Department of Genetics, College of Life Sciences and the Institute for Advanced Studies, Wuhan University, Wuhan, Hubei 430072, China; 2 National Laboratory of Biomacromolecules, CAS Center for Excellence in Biomacromolecules, Institute of Biophysics, Chinese Academy of Sciences, Beijing 100101, China; 3 The Department of Molecular and Human Genetics, Baylor College of Medicine, One Baylor Plaza, Houston, TX 77030, USA

## Abstract

Repair of DNA double-strand breaks (DSBs) requires eviction of the histones around DNA breaks to allow the loading of numerous repair and checkpoint proteins. However, the mechanism and regulation of this process remain poorly understood. Here, we show that histone H2B ubiquitination (uH2B) promotes histone eviction at DSBs independent of resection or ATP-dependent chromatin remodelers. Cells lacking uH2B or its E3 ubiquitin ligase Bre1 exhibit hyper-resection due to the loss of H3K79 methylation that recruits Rad9, a known negative regulator of resection. Unexpectedly, despite excessive single-strand DNA being produced, *bre1Δ* cells show defective RPA and Rad51 recruitment and impaired repair by homologous recombination and response to DNA damage. The HR defect in *bre1Δ* cells correlates with impaired histone loss at DSBs and can be largely rescued by depletion of CAF-1, a histone chaperone depositing histones H3-H4. Overexpression of Rad51 stimulates histone eviction and partially suppresses the recombination defects of *bre1Δ* mutant. Thus, we propose that Bre1 mediated-uH2B promotes DSB repair through facilitating histone eviction and subsequent loading of repair proteins.

## INTRODUCTION

DNA repair is crucial for faithful transmission of genetic information into daughter cells. DNA double-strand breaks (DSBs) are potent cytotoxic DNA lesion challenging genome stability that must be repaired faithfully to prevent cell death or tumorigenesis (1,2). DSBs can be repaired by either homologous recombination (HR) or non-homologous end joining (NHEJ), and the choice between these two pathways is regulated by the cell cycle (3,4). HR is the dominant repair pathway in S and G2 phases. It requires a homologous template to direct the repair and is considered to be more accurate (3,4). NHEJ operates predominantly in the G1 phase when sister chromatids are not available for repair. NHEJ is less accurate as it can lead to small insertion or deletions at DSBs (3,4).

A crucial step that favors HR while discriminates against NHEJ is the resection of 5′-ends at DSBs that generates 3′-end single-strand DNA (ssDNA). It is initiated by the Mre11–Rad50–Xrs2 (MRX) complex in yeast (MRE11–RAD50–NBS1 complex in mammals) in cooperation with the Sae2 (CtIP in mammals) protein (5–7). Long-range resection is carried out by Exo1 or Sgs1-Dna2, two partially redundant pathways (7,8). Exposure of 3′-end ssDNA recruits the ssDNA binding protein complex RPA. Mec1-Ddc2 complex is recruited to RPA bound ssDNA to activate DNA damage checkpoint. RPA is subsequently replaced by the recombinase Rad51 to form long nucleoprotein filament that carries out homology search and strand invasion (9). Besides RPA, Rad51 and Mec1-Ddc2, a large number of other proteins involved in checkpoint signaling or damage repair are also heavily loaded at resected DSB ends. Particularly, some of them are loaded with a high local concentration so they form strong nuclear foci at the site of DNA lesion (10). Therefore, a key question is how these proteins are assembled within such a limited range of DSB end that is occupied by histones.

The chromatin structure alteration at the lesion sites is believed to provide access for the DNA damage response and repair proteins. A number of studies have shown that during HR histones around DSBs are partially evicted in a resection-dependent manner (11–16). This coincides with the observation that chromatin around DSBs shows increased susceptibility to micrococcal nuclease or restriction digestion (13,17). As a consequence, defective histone eviction has been linked to delayed Rad51 recruitment and repair (13). Histone eviction occurs in a fashion coupled to resection and appears to be affected by several ATP-dependent chromatin remodeling complexes (11–14,17). Since these remodelers also promote resection, it is indiscernible whether resection itself is enough to drive histone disassembly during HR. Moreover, how histone eviction is regulated *in vivo* and what is the impact of its deregulation on HR remain poorly understood.

The evolutionarily conserved histone H2B mono-ubiquitination (uH2B) has been shown to disrupt ordered chromatin structure, creating a relaxed chromatin (18). H2B ubiquitination occurs on the residue lysine 123 (K123) in budding yeast, equivalent to K119 in *Schizosaccharomyces pombe* and K120 in mammals. This modification is catalyzed by the E2 ubiquitin conjugating enzyme Rad6 (Ubc6 or RAD6 in human) in cooperation with the E3 ubiquitin ligase Bre1 in yeast (RNF20/RNF40 in mammals) (19–22), and is essential for the di- and tri-methylation of histone H3 on K4 and K79 (20,23–25). uH2B is a mark associated with transcriptional activation, and its establishment requires the PAF complex and the histone chaperone FACT during transcription (26–29). uH2B can cooperate with FACT to facilitate histone assembly in transcription. It can also function in nucleosome destabilization and H2A/H2B dimer removal during transcription elongation (30–32). Moreover, this modification is present at the replication origin where it stimulates replication fork progression and stabilizes replisome upon replication stress (33). In addition, uH2B also plays important roles in meiosis and in centromere and telomere maintenance (34–38). Finally, uH2B has been implicated in DSB repair in *S. pombe* and mammals (39–43). In yeast, the absence of its E3 ligase Bre1 results in increased susceptibility to ionizing radiation (IR) (44–46). The molecular mechanism by which uH2B regulates DSB repair has not been fully understood.

In this study, we provide evidence that the Bre1-dependent uH2B plays a key role in stimulating histone eviction from ssDNA at DSB ends in a fashion independent of resection or the ATP-dependent chromatin remodelers. This histone loss facilitates the subsequent loading of recombination proteins and repair by HR.

## MATERIALS AND METHODS

### Yeast strains and plasmids

Strains used in this study are derivatives of JKM139 (*ho MAT****a****hml::ADE1 hmr::ADE1 ade1-100 leu2-3,112 trp1::hisG’ lys5 ura3-52 ade3::GAL::H*O) or tGI354 (*MATa-inc arg5,6::MATa-HPH ade3::GAL::HO hmr::ADE1 hml::ADE1 ura3-52*). All yeast strains are listed in Supplemental Table 1. Yeast mutant strains were generated with standard genetic manipulation. The sequences of all oligonucleotide primers used are available upon request.

### Fluorescence microscopy

Live cells with or without phleomycin treatment were examined using an Olympus BX53 fluorescence microscope with a 100× oil immension objective lens and an YFP filter. Fluorescent images were captured by using an Olympus DP80 digital camera. Images were processed using Olympus Cellsens software. The percentage of cells carrying Rad52-YFP foci was calculated after analyzing three independent experiments. Approximately 200 cells were counted for each experiment.

### Chromatin immunoprecipitation (ChIP)

Exponentially growing cells (1.2 × 10^7^ cells/ml) in YEP-Raffinose medium were subject to DSB induction by addition of 2% galactose. Samples were collected at indicated time points. ChIP assays were carried out as previously described (11). DNA shearing was performed on a Diagenode Bioruptor. Antibodies used were anti-Myc (MBL), anti-FLAG (Cell Signaling Technology) and anti-HA (MBL). Purified DNA were analyzed by real-time quantitative PCR with primers that specifically anneal to DNA sequences located at indicated distances from the DSB using the following conditions: 95°C for 10 min; 40 cycles of 95°C for 15 s, 60°C for 1 min, and 72°C for 30 s. Histone loss was measured by ChIP-qPCR using the strains carrying FLAG-tagged H3 according to previously described method (11).

### Western Blotting

Whole cell extracts were prepared using a trichloroacetic acid (TCA) method as previously described (11). Samples were resolved on an 8% or 12% SDS-PAGE gel and transferred onto a PVDF membrane (Immobilon-P; Millipore) using a semi-dry method. Anti-Myc and anti-HA antibody antibodies were purchased from MBL. Anti-FLAG antibody for Western blot was obtained from Sigma (F1804). Anti-mouse and rabbit IgG HRP-conjugated secondary antibodies were purchased from Santa Cruz Biotechnology. Blots were developed using the Western Blotting substrate (Bio-Rad).

### Analysis of resection kinetics at DSB ends

Resection of DSB ends was analyzed at an HO endonuclease-induced DSB at the *MAT* locus on chromosome III using Southern blots as previously described (7,11). Galactose induction, sample collection, DNA isolation and purification were carried out as described by Chen *et al.* (11). Purified DNA was digested with EcoRI and separated on a 0.8% agarose gel followed by transferring onto a nylon membrane. Radiolabeling of DNA probes was performed following manufacturer's instruction (Takara). Southern blotting and hybridization with radiolabeled DNA probes was performed as described previously (7,11). The blot was exposed in a Phosphor screen. Signal on the screen was captured by scanning in an OptiQuant Cyclone Plus machine (Perkin Elmer). Intensities of target bands were analyzed with OptiQuant software (Perkin Elmer) and normalized to the TRA1 probe. Resection rate was calculated as previously described (7). Three independent experiments were performed for each strain.

### Ectopic recombination and Single-strand annealing (SSA)

To test the viability for ectopic recombination or SSA, cells were grown in pre-induction medium (YEP-Raffinose) overnight to early log phase. Cells were then diluted to a concentration of ∼1 × 10^3^ cells/ml. Approximately 200 cells were plated on each YEPD or YEP-Gal plate. Plates were incubated at 30°C for 3–5 days. Viability (%) = (the number of colonies grown on YEP-Gal)/(the number of colonies grown on YEPD) × 100%. At least three independent experiments were performed for each strain. Southern blot was performed as previously described (47). To measure the repair kinetics for ectopic recombination, we quantified and normalized the pixel intensity of target bands to that of corresponding parental bands on blots. The resulting values were further normalized to that of the control sample (uncut).

### Drug sensitivity test

Yeast cells were grown in YEPD rich medium overnight to saturation. Undiluted cell culture and 1/10 serial dilutions of each cell culture were spotted onto YPD plates containing different DNA-damaging agents at indicated concentrations. Plates were incubated at 30°C for 3 days before analysis.

### Gene targeting assay

Gene targeting was performed as described previously with modifications (11). A circular plasmid pRS316 and a linear DNA cassette containing a *URA3* gene in the center and a 1.5 kb fragment at each side that corresponds to the flanking sequences of the endogenous *ISU1* gene was separately transformed into equal amount of cells and were plated on SD-Ura medium. HR allows integration of the cassette into genome thereby enabling cells to grow on medium without uracil. Plates were incubated at 30°C for three to five days and colonies were counted. A relative targeting efficiency was calculated by dividing the number of colonies derived from transformation with linear cassette by the number of colonies derived from transformation with circular plasmid. At least four independent experiments were performed for each strain.

### Chromatin fraction assay

Log phase cells were treated with 20 μg/ml of phleomycin for 2 hrs. Collected cells were washed with sterile H_2_O and then resuspended in 900 μl of cold sorbitol solution (1 M sorbitol, 50 mM Tris–HCl pH 7.5). Cells were subject to lyticase (Sigma) digestion for 1 h at 37°C followed by centrifugation at 12 000 × g for 2 min at 4°C. The pellet was washed with sorbitol solution and resuspended in 400 μl of lysis buffer (0.5 mM spermidine, 1 mM β-mecaptoethanol, 0.1% NP40, 50 mM NaCl, 10 mM Tris–HCl pH 8.0, 5 mM MgCl_2_, 5 mM CaCl_2_) supplied with protease inhibitors. Samples were incubated at 4°C with rotation for 1h. Half of each sample was stored as total extract while the other half of each sample was centrifuged at 12 000 × g for 2 min at 4°C. The supernatant was stored as the soluble fraction. The pellet was washed with lysis buffer and resuspended in 200 μl of lysis buffer as the chromatin fraction. 2× SDS protein loading buffer was added to each fraction and the mixture was boiled at 100°C for 5 min. Protein distribution in each fraction was detected by western blot using the antibodies against FLAG (Sigma), GAPDH (GeneTex) or H3 (Abclonal).

### Isolation of total RNA and RNA-seq

The WT cells (JKM139) and the isogenic *bre1Δ* mutant cells grown in YEP-Raffinose medium to log phase were collected for RNA extraction. Isolation of total RNA was performed following the method described previously (11). Construction of the sequencing library and RNA-seq were carried out at Shanghai Ouyi Biotech Company. Two independent experiments were carried out for each strain and the results were combined. The original RNA-seq data was deposited at GenBank under the SRA accession no. SRP131704.

## RESULTS

### The ubiquitin E3 ligase Bre1 promotes DNA damage response

**Figure 1.**
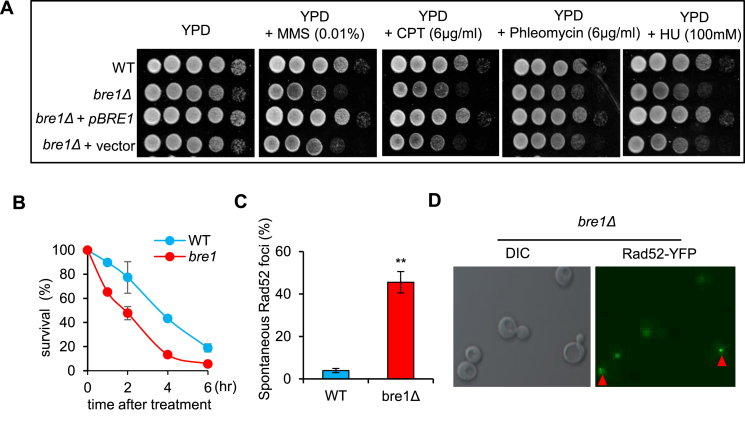
Bre1 stimulates proper DNA damage response and repair. (**A**) DNA damage sensitivity test for indicated strains. 10-fold serial dilutions of indicated cultures on plates with various DNA damaging-agents at indicated concentrations. Plates were incubated at 30°C for 3–4 days. (**B**) Survival curve for the WT and *bre1Δ* mutant cells following acute MMS treatment (0.1%). (**C, D**) Microscopy analysis and quantification of spontaneous Rad52-YFP focus formation in the WT and *bre1Δ* cells. A representative image is presented. Error bar represents standard deviation from at least three independent experiments. ** denotes statistical significance *P* < 0.01 (*t*-test).

Previous genetic studies suggested that the E3 ubiquitin ligase Bre1 is required for proper resistance to IR in yeast (45). In line with previous studies, we observed that Bre1 deficient cells are susceptible to a variety of DNA damaging-agents, including camptothecin (CPT), methyl methanesulfonate (MMS), phleomycin and hydroxyurea (HU) (Figure 1A). The sensitivity was fully rescued by ectopic expression of a *BRE1* gene. Furthermore, *bre1Δ* mutant displayed a reduced survival rate upon acute MMS (0.1%) treatment as compared to the wild type (WT) cells (Figure 1B). Therefore, Bre1 is needed to respond to both acute and chronic genotoxic stresses. Notably, Bre1 is also important to prevent or repair spontaneous DNA damage, since *bre1Δ* mutant cells accumulated ten time more spontaneous Rad52-YFP foci than the WT cells (∼4%) (Figure 1C and D). This difference was not caused by cell cycle shift, since deletion of *BRE1* did not alter the cell-cycle distribution (Supplementary Figure S1). Thus, the ubiquitin E3 ligase Bre1 plays an important role in response to both induced and spontaneous DNA damages.

### Bre1 plays an important role in DSB repair by homologous recombination

**Figure 2.**
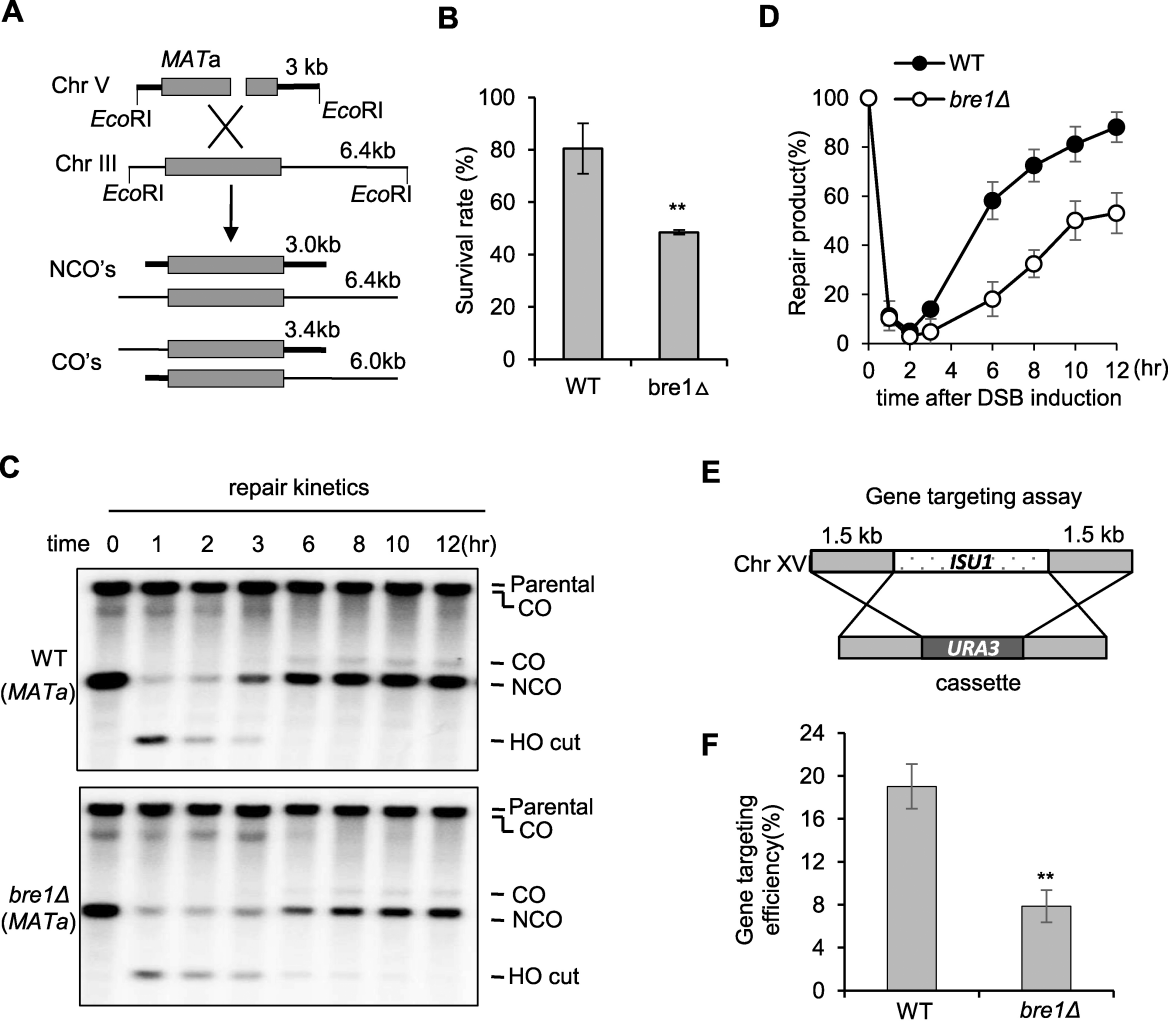
Bre1 promotes DSB repair by HR. (**A**) Scheme showing an ectopic recombination system. CO: crossover; NCO: non-crossover. (**B**) Survival rate for the WT and *bre1Δ* mutant cells repaired by ectopic recombination. (**C, D**) Southern blot analysis and quantification of repair kinetics for WT and *bre1Δ* cells. (**E**) Scheme showing the gene targeting assay. Homology between the flanking sequences at both sides of the *URA3* gene on the cassette and *ISU1* gene on chromosome XVI allows the integration of *URA3* gene into the genome by HR. (**F**) Plot showing relative gene targeting efficiency for WT and *bre1Δ* strains. Error bar represents standard deviation from at least three independent experiments. ** denotes statistical significance *P* < 0.01 (*t*-test).

Rad52 protein is crucial for repair by HR. To directly assess whether Bre1 plays a role in HR, we employed an ectopic recombination system in which a DSB generated by the HO endonuclease within the *MATa* sequence on chromosome V is repaired by HR with the *MATa*-inc sequence on chromosome III as a donor (Figure 2A) (48). Over 80% of WT cells have successfully completed the repair and survived, while only ∼47% of *bre1Δ* mutant cells survived (Figure 2B). Consistently, the mutant repaired the break with a slower kinetics than the WT cells (Figure 2C and D). However, the ratio of crossover recombination appeared to remain unaffected in this mutant (Supplementary Figure S2). The repair defect in *bre1Δ* mutant was unrelated to DSB repair by NHEJ, since depletion of the core NHEJ protein Yku70 did not affect the repair rate in both WT and *bre1Δ* cells (Supplementary Figure S3). To verify the role of Bre1 in HR, we employed a gene targeting assay to measure HR efficiency (Figure 2E). A similar defect was observed for *bre1Δ* mutant (Figure 2F). Together, these results demonstrate that Bre1 is important for DSB repair by HR.

### Bre1 prevents hyper-resection of DSB ends

**Figure 3.**
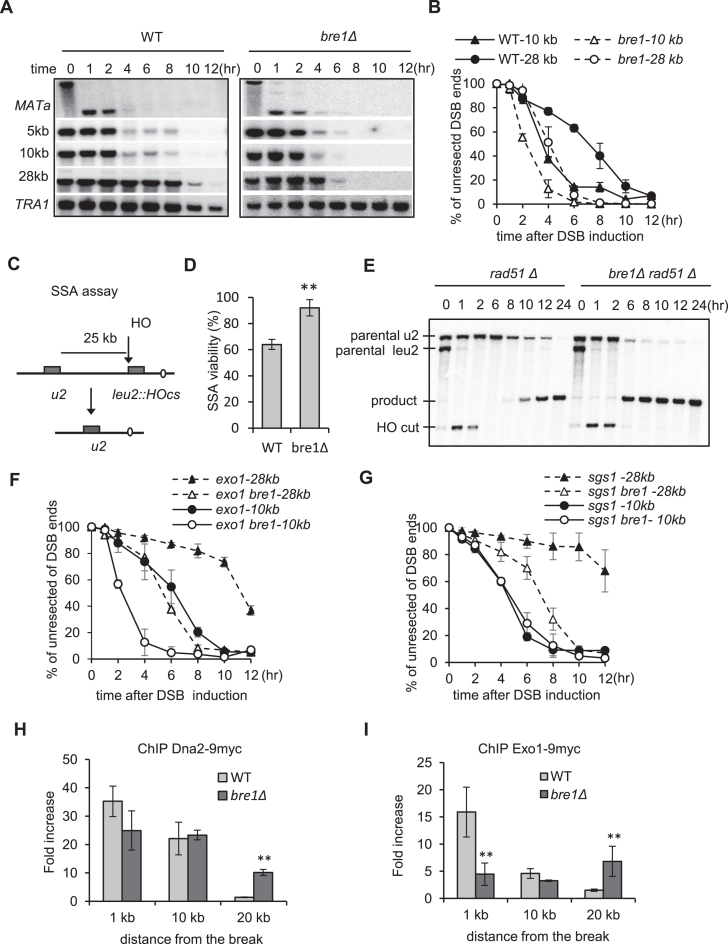
Bre1 suppresses hyper-resection of DSB ends. (**A, B**) Southern blot analysis and quantification of resection kinetics for WT and *bre1Δ* cells. (**C**) A diagram showing the single-strand annealing (SSA) assay between two partial *leu2* gene repeats separated by 25 kb. Rad51 was deleted to prevent repair by alternative HR pathways. (**D**) Survival rate for DSB repair by SSA. (**E**) Southern blot showing SSA repair kinetics in WT and *bre1Δ* mutant. (**F**, **G**) Quantification of resection kinetics for indicated strains. The corresponding Southern blot is presented in in Supplementary Figure S4. (**H, I**) ChIP-qPCR analysis for the recruitment of Dna2-9xmyc and Exo1-9xmyc at 4 h following DSB induction in WT and *bre1Δ* mutant at indicated locations. Error bar denotes standard deviation from three independent experiments. ** *P* < 0.01 (*t*-test).

A crucial step during HR is the processing of the 5′-ends of DSBs by resection enzymes to generate ssDNA. We asked whether end processing was affected in *bre1Δ* mutant. To this end, we employed a haploid yeast strain in which the HO endonuclease generates a single non-repairable DSB at the *MAT* locus on chromosome III upon galactose induction. Repair by HR is prevented because the donor sequences *HML* and *HMR* were deleted (7,11). We monitored the resection kinetics for the WT and *bre1Δ* mutant cells by Southern blot. In the WT cells, resection proceeded at a rate of ∼ 3kb/hr, close to that observed previously (7) (Figure 3A and B). The *bre1Δ* mutant exhibited a normal resection speed at sites proximal to the break. In contrast, the long-range resection became much faster in this mutant as measured at 10 or 28 kb from the break (Figure 3A and B). To verify this result, we compared their efficiencies for DSB repair by single-strand annealing (SSA) (49). In this assay, resection up to 25-kb distance is essential to allow the annealing between two partial *leu2* repeats (Figure 3C). Rad51, which is required for typical HR but is dispensable for SSA, was deleted to prevent alternative repair pathways. Approximately 60% of WT cells survived, while about 90% of *bre1Δ* cells successfully completed the repair (Figure 3D). Accordingly, the mutant cells repaired the break with a faster kinetics (Figure 3E). Together, these results demonstrate that Bre1 plays an important role in preventing hyper-resection. Since DSB repair by SSA operates efficiently, it is likely that the HR defect of *bre1Δ* mutant is limited to Rad51-dependent events.

In WT cells, long-range resection depends on two parallel pathways mediated by Sgs1/Dna2 and Exo1. We found that the hyper-resection in *bre1Δ* mutant was also carried out by these two pathways but not by any other unknown enzymes since simultaneously deletion of *SGS1* and *EXO1* in *bre1Δ* cells nearly abolished the resection at *MAT* locus, and the defect was comparable to that seen in *sgs1Δ exo1Δ* double mutant (Supplementary Figure S4). In either *exo1Δ* or *sgs1Δ* single mutant, additional deletion of *BRE1* greatly accelerated long-range resection (Figure 3F, G and Supplementary Figure S4), indicating that Bre1 restrains hyper-resection by both pathways. Accordingly, we found that both Dna2 and Exo1 were recruited to sites as far as 20 kb from the break in *bre1Δ* mutant at 4 h whereas they were recruited only to sites relatively proximal to the break (within 10 kb) in the WT cells (Figure 3H–I), suggesting that the enzymes spread along chromatin with a faster kinetics in the mutant. The levels for these proteins are comparable between the WT and *bre1Δ* cells (Supplementary Figure S5). Moreover, we noted that the double mutant *bre1Δ sgs1Δ* but not *bre1Δ exo1Δ* were significantly more sensitive to DNA damaging agents than the respective single mutants (Supplementary Figure S6). Thus, Bre1 and Sgs1 may act additively in response to DNA damages.

### Bre1 is directly recruited to DSBs

**Figure 4.**
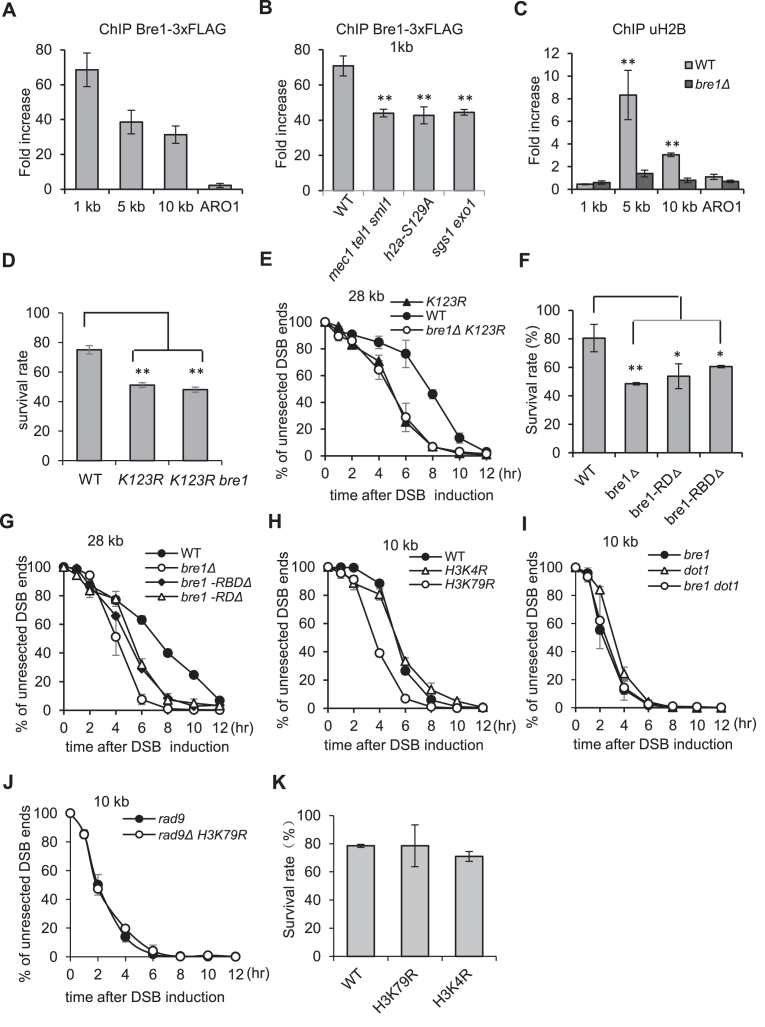
Bre1-dependent uH2B regulates resection and repair by HR. (**A**, **B**) ChIP-qPCR analysis of Bre1-3xFLAG recruitment at indicated locations 4 h after DSB induction in indicated strains. *ARO1* serves as a control locus where there is no DSBs. (**C**) ChIP-qPCR showing the enrichment of uH2B at different locations 4 h after DSB induction in WT and *bre1Δ* mutant cells. (**D**) Plot showing ectopic recombination rate for indicated strains. (**E**) Quantification of resection kinetics for indicated strains. (**F**) Plot showing ectopic recombination efficiency for WT cells and indicated *bre1* mutants. (**G–J**). Quantification of resection kinetics for indicated strains. The Southern blot for resection is presented in Supplementary Figure S4. (**K**) Plot showing ectopic recombination rate for indicated strains. Error bar means standard deviation from at least three independent experiments. Asterisks denote statistical significances. *P < 0.05 **P < 0.01 (*t*-test). The recombination assay employed in this figure is as illustrated in Figure 2A.

Previous global gene expression analyses revealed that deletion of *BRE1* does not alter the expression of genes involved in DNA repair (46). This is confirmed by our RNA-seq analysis (Supplemental Table 2). To examine whether Bre1 plays a direct role in DSB repair, we tested Bre1-3xFLAG recruitment at DSBs by chromatin immunoprecipitation (ChIP). Bre1-3xFLAG was robustly enriched at sites proximal to the ends or at 5 or 10 kb away at 4 h, but not at the control locus *ARO1* which is not linked to DSB (Figure 4A). Bre1 recruitment was only partially decreased in *mec1Δ tel1Δ sml1Δ, h2a-S129A* or *sgs1Δ exo1Δ* mutant cells that are deficient either in checkpoint response or resection. Thus, Bre1 recruitment is partially dependent on checkpoint and resection (Figure 4B). Bre1 is a known E3 ligase involved in H2B ubiquitination (19). To test whether Bre1 was active at DSBs, we measured the levels of uH2B at DSB ends by ChIP. As expected, we observed a significant enrichment of uH2B at 5 and 10 kb locations at 4 h in the WT cells but not in *bre1Δ* mutant (Figure 4C). Thus, Bre1 is directly recruited to DSBs to stimulate local uH2B enrichment.

### Bre1 acts through H2B ubiquitination to regulate DSB repair

To test whether the role of Bre1 in DSB repair is achieved through affecting H2B ubiquitination, we assessed DSB repair by ectopic recombination for the WT cells and the isogenic *H2B-K123R* and *bre1Δ H2B-K123R* mutant cells. K123R mutation abolished H2B ubiquitination on this residue. 78% of the WT cells completed the repair and survived (Figure 4D). In contrast, only 46% of *K123R* mutant cells survived (Figure 4D), as seen for *bre1Δ* single mutant. Importantly, additional deletion of *BRE1* in *K123R* cells did not further reduce the viability (Figure 4D). Consistently, the repair kinetics was slower in *K123R* or *bre1Δ K123R* mutant than in the WT cells (Supplementary Figure S7). Altogether, these results indicate that the role of Bre1 in HR is executed through uH2B.

To test whether the role of Bre1 in suppressing resection is acting through uH2B, we measured the resection kinetics for the WT and *K123R* mutant cells. As expected, resection became much faster in *K123R* mutant, as seen in *bre1Δ* mutant cells (Figures 3A, B and 4E). Moreover, additional deletion of *BRE1* in *K123R* mutant did not further increase the resection rate (Figure 4E and Supplementary Figure S4), indicating that Bre1 restrains resection via regulating uH2B. Finally, upon CPT, MMS or HU treatment, *K123R* cells exhibited a similar resistance profile as observed in *bre1Δ* or *bre1ΔK123R* mutants (Supplementary Figure S8). Together, these results demonstrate that the functions of Bre1 in DNA damage response, resection and repair by HR are executed through controlling H2B-K123 ubiquitination.

### The E3 ligase activity is important for Bre1’s function in DSB repair

Bre1 possesses a RING domain (RD) and a Rad6-binding domain (RBD), both contributing to its ubiquitin E3 ligase activity (21,50). To evaluate the importance of its ligase activity in DNA damage response, we generated *bre1* mutants lacking either of the two domains. We found that both mutants were susceptible to CPT and MMS, to an extent as seen in *bre1Δ* mutant (Supplementary Figure S9). Furthermore, we measured the repair by ectopic recombination for these mutants. Compared to the WT cells, only 52% of *bre1-RDΔ* mutant cells completed the repair (Figure 4F), underscoring the importance of the RING domain in HR; while *bre1-RBDΔ* cells exhibited a slightly higher viability (60%) than *bre1Δ* mutant (Figure 4F), which may result from its residual ligase activity (50). Next, we analyzed their resection rates and found that both mutants exhibited a faster resection than the WT cells (Figure 4G and Supplementary Figure S4). Together, these results indicate that the E3 ligase activity is critical for Bre1 to respond to DSBs.

### Bre1-mediated uH2B regulates resection through permitting H3K79 methylation

Bre1-dependent uH2B is essential for di- and tri-methylation of histone H3 on K4 and K79 (23–25,51,52). To examine whether the role of uH2B in DSB repair was relevant to H3K4 or H3K79 methylation, we analyzed resection for *H3K4R* and *H3K79R* point mutants that are deficient in methylation on these sites. *H3K4R* mutation exhibited a normal resection rate as seen in WT cells. In contrast, *H3K79R* mutant displayed a much faster resection, as seen in *K123R* or *bre1Δ* mutant cells (Figures 3A, B and 4H, Supplementary Figure S4). Deletion of *DOT1*, the methyltransferase for H3K79 methylation, impaired the chromatin recruitment of Rad9, a known resection barrier, leading to hyper resection (11,53). Both *dot1Δ* and *bre1Δ* single mutants and *bre1Δ dot1Δ* double mutant exhibited a comparable resection kinetics (Figure 4I and Supplementary Figure S4), indicating that Bre1-mediated uH2B regulates resection through controlling the methylation of H3K79 but not H3K4. Furthermore, we noted that additional H3K79R mutation in *rad9Δ* cells did not further accelerate the resection (Figure 4J and Supplementary Figure S4), suggesting that the effect of loss of H3K79 methylation on resection is through affecting Rad9.

### The HR repair function of uH2B is independent of H3K4 or H3K79 methylation or ATP-dependent chromatin remodelers

Next, we examined whether the repair function of Bre1 is related to H3K4 or H3K79 methylation. We found that both *H3K4R* and *H3K79R* mutants exhibited a normal repair rate and kinetics in ectopic recombination, as observed in the WT cells (Figure 4K and Supplementary Figure S7). Consistently, *HK123R* mutant but not *H3K4R* or *H3K79R* mutant displayed significant sensitivities to CPT, phleomycin and MMS (Supplementary Figure S10). Although uH2B acts through Dot1-mediated H3K79 methylation to suppress resection, we noted that *dot1Δ* single mutant was not sensitive to these drugs, and *bre1Δ* single mutant and *bre1Δ dot1Δ* double mutant exhibited a similar resistance pattern in response to these drugs (Supplementary Figure S10). These results indicate that the repair function of Bre1 and uH2B in HR is independent of H3K4 or Dot1-mediated H3K79 methylation.

Several ATP-dependent chromatin remodeling complexes including INO80, RSC, Fun30 and SWR have been reported to stimulate resection of DSB ends (11,12,14,17,54). However, unlike *bre1Δ* mutant, deletion of each of these remodelers did not impair the eventual repair efficiency by ectopic recombination (Supplementary Figure S11), suggesting that the repair function of uH2B in HR is independent of these chromatin remodelers. Indeed, we found that the recruitment of these remodelers remains unaffected in *bre1Δ* mutant (Supplementary Figure S11).

### Bre1-dependent uH2B promotes the recruitment of RPA and Rad51

**Figure 5.**
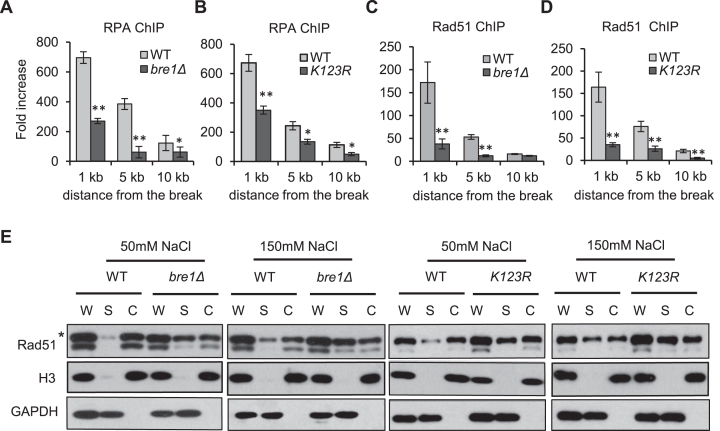
Bre1-dependent uH2B promotes RPA and Rad51 recruitment at DSBs. (**A, B**) ChIP-qPCR analysis of RPA-3xFLAG enrichment at 1, 5 or 10 kb locations in WT, *bre1Δ* and *K123R* cells 4 h after DSB induction. (**C, D**) ChIP-qPCR analysis of Rad51-3xFLAG enrichment at indicated locations in WT, *bre1Δ* and *K123R* cells 4hr after DSB induction. Error bar means standard deviation from three independent experiments. *P < 0.05, **P < 0.01 (*t*-test). (**E**) Chromatin fraction and Western blot analysis of Rad51 association with chromatin in indicated strains after phleomycin treatment. W: whole cell extracts; S: soluble fraction; C: insoluble chromatin fraction. The asterisk marks full-length Rad51.

Next, we tested whether the key recombination proteins RPA and Rad51 were properly recruited in the absence of uH2B. As expected, both RPA and Rad51 were recruited robustly to DSB in the WT cells at 4hr following DSB induction, with a distribution ranging from 1 to 10 kb from the break (Figure 5A–D). However, the recruitment for both proteins was significantly decreased in either *bre1Δ* or *K123R* mutant (Figure 5A–D). These defects were not caused by any alterations in protein abundance since RPA and Rad51 were expressed at comparable levels between the WT and *bre1Δ* mutant cells (Supplementary Figure S5). Also, we excluded the possibility that the defect was related to a faster resection, since both proteins were efficiently recruited in *dot1Δ* mutant which phenocopies *bre1Δ* mutant in resection (Supplementary Figure S12 and Figure 4J). To test whether this phenomenon is locus-specific, we performed chromatin fraction assay to examine the global association of Rad51 on chromatin following phleomycin treatment. In the WT cells, Rad51 predominantly retained on chromatin in the presence of low concentration of salt, and it became partially disassociated from chromatin at increased salt condition (Figure 5E). However, in *bre1Δ* or *K123R* cells, we detected significantly more Rad51 in the soluble fraction at both conditions (Figure 5E). Thus, Bre1-dependent uH2B is important for the recruitment or retention of Rad51 on ssDNA on damaged chromatin.

### uH2B facilitates histone loss at DSB ends

Given that ssDNA formation was proficient while RPA and Rad51 enrichment was defective in *bre1Δ* or *K123R* mutant, we reasoned that uH2B may control unidentified steps that are required for proper RPA and Rad51 loading. We first explored whether uH2B may directly recruit Rad51 by testing *in vitro* Rad51 binding with uH2B or nucleosome octamers assembled with uH2B. However, we failed to observe any noticeable interaction between them (data not shown), suggesting that Rad51 does not directly interact with uH2B.

**Figure 6.**
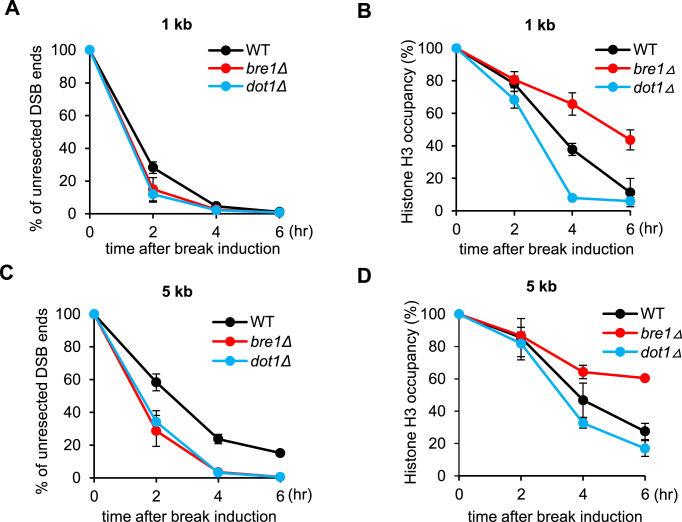
Bre1 stimulates histone loss following resection. (**A**,**C**) Quantification of resection kinetics at 1 kb or 5 kb location for indicated strains. The corresponding Southern blot is presented in Figure 3A and S4. (**B**, **D**) ChIP-qPCR analysis of histone H3 occupancy at 1 or 5 kb location for WT cells and *bre1Δ* or *dot1Δ* mutants. Plotted values are mean value from three independent experiments. Error bar represents standard deviation.

Previous studies revealed that histones interfere with the association of HR protein with ssDNA and defective histone eviction at DSBs has been linked to delayed Rad51 recruitment (13,55). Since uH2B is known to be able to disrupt compacted chromatin and affect nucleosome dynamics on chromatin (18,31,33), we thus hypothesized that decreased loading of RPA and Rad51 in *bre1Δ* mutant may be relevant to an altered histone dynamics at DSB ends. To test this, we evaluated histone occupancy in the WT and *bre1Δ* mutant cells by ChIP-qPCR as described (11). As previously noted, histone H3 loss occurred following DSB end resection in the WT cells at both 1 kb and 5 kb locations (Figure 6A–D). In contrast, H3 loss was significantly delayed in *bre1Δ* cells at both locations although resection was faster in this mutant (Figure 6A–D). This delay was not due to the fast resection, since H3 loss was much faster in *dot1Δ* cells which phenocopy *bre1Δ* mutant in resection (Figure 6A–D). Similarly, H3 loss was also significantly delayed in *K123R* mutant (Supplementary Figure S13). These results indicate that resection itself is not sufficient to drive histone eviction, while the Bre1-dependent uH2B plays an important role in stimulating histone disassembly or loss from ssDNA following resection. In support of this result, it has been reported that histones can interact efficiently with ssDNA and form nucleosome-like structure (56–58). Thus, the defects of *bre1Δ* mutant in RPA and Rad51 loading and in repair by HR are likely resulted from the retention of histones on ssDNA.

CAF-1 (chromatin assembly factor 1) is a key H3–H4 chaperone involved in histone deposition in DNA replication or repair (59). Deletion of *CAC1*, which encodes a subunit of CAF-1 complex, resulted in mild defects in DSB repair by ectopic recombination and reduced DNA damage resistance (Supplementary Figure S14). We found that deletion of *CAC1* significantly rescued the defect of *bre1Δ* cells in HR repair (Supplementary Figure S14). Interestingly, the double mutant *cac1Δ bre1Δ* showed enhanced resistance to DNA damages compared to *cac1Δ* single mutant (Supplementary Figure S14). The genetic interaction between Bre1 and CAF-1 further supports a role of Bre1 in promoting histone loss on DNA breaks.

### Overexpression of *RAD51* accelerated histone eviction and improved HR repair in *bre1Δ* cells

**Figure 7.**
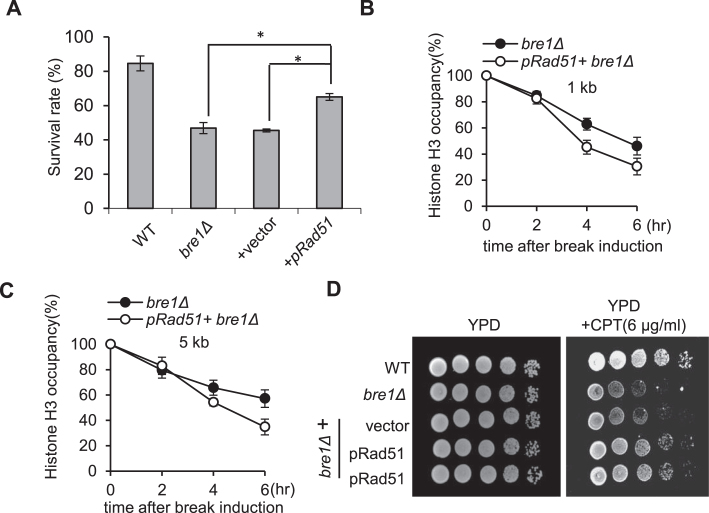
Overexpression of *RAD51* stimulates histone loss at DSBs and improves HR efficiency in *bre1Δ* mutant. (**A**) Plot showing ectopic recombination rate for indicated strains. * statistical significance (*P* < 0.05). (**B, C**) ChIP-qPCR analysis of histone H3 occupancy at 1 and 5 kb locations for *bre1Δ* mutant carrying an empty vector or the pRS426-Rad51 plasmid. (**D**) Spotting assay for 10-fold serial dilutions of indicated strains on YPD plate with or without CPT.

Given that histones and HR proteins compete for binding to ssDNA and defective histone loss can interfere with Rad51 recruitment (13,55), we wondered whether overexpression of Rad51 can accelerate histone eviction in *bre1Δ* mutant and improve HR. Therefore, we transformed a high-copy plasmid carrying *RAD51* gene into *bre1Δ* mutant and examined the efficiency of ectopic recombination. Overexpression of Rad51 partially restored DSB repair efficiency in *bre1Δ* mutant cells (Figure 7A). Next, we tested whether excessive Rad51 could impact the histone loss in *bre1Δ* mutant cells. Indeed, excess amount of Rad51 significantly accelerated histone eviction at 1 and 5 kb location in the mutant cells (Figure 7B, C). Furthermore, overexpression of Rad51 augmented the resistance of *bre1Δ* cells to CPT (Figure 7D). These results indicate that histone retention on chromatin impedes Rad51 loading and repair by HR, while uH2B plays an important role in stimulating histone loss thereby facilitating HR.

## DISCUSSION

Histone H2B ubiquitination is an evolutionarily conserved post-translational modification in eukaryotes. It plays critical roles in transcription, DNA replication and repair, centromere and telomere maintenance and in meiosis. In this study, we provide evidence that the E3 ubiquitin ligase Bre1 directly acts at DSBs to stimulate local H2B ubiquitination, by which Bre1 promotes DSB repair by HR through stimulating histone eviction and subsequently the loading of recombination proteins at DSB ends.

**Figure 8.**
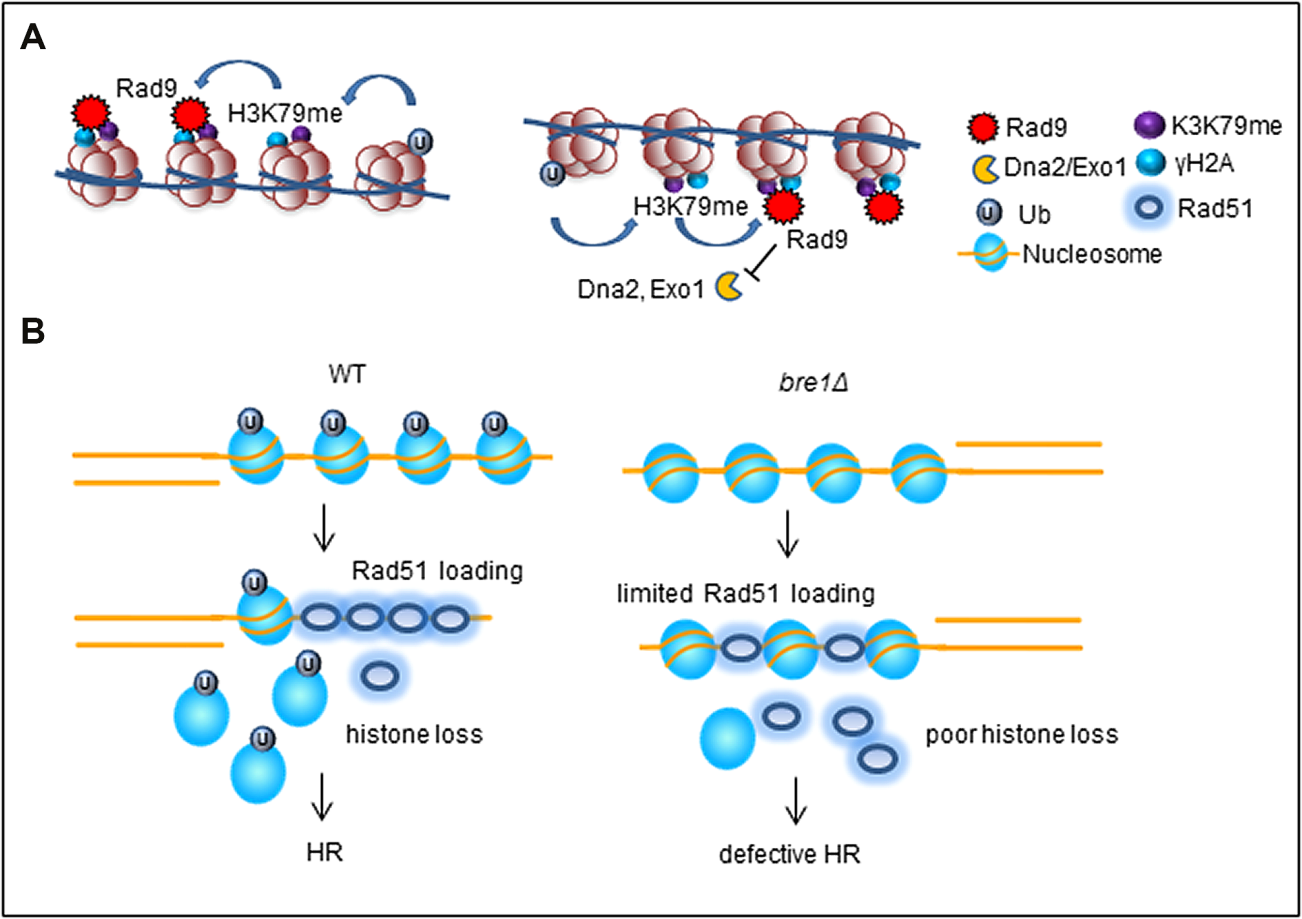
A working model showing the function of Bre1-dependent uH2B in HR. (**A**) uH2B permits H3K79 methylation and the subsequent recruitment of Rad9, a known negative regulator that suppresses long-range resection by Sgs1/Dna2 and Exo1 pathways. Thus, uH2B acts to prevent hyper-resection of DSB ends. (**B**) After resection, histones at DSB ends are partially evicted, which facilitates Rad51 loading and repair by HR. uH2B is required to stimulate histone eviction at DSB ends. In *bre1Δ* mutant, the retention of histones on ssDNA impedes Rad51 binding, leading to defective HR.

Bre1-dependent uH2B at least executes two layers of independent regulations. First, it prevents hyper-resection through permitting H3K79 methylation and Rad9 recruitment, which is known to suppress long-range resection carried out by Sgs1/Dna2 and Exo1 (Figure 8A). Second, uH2B stimulates histone eviction at DSB ends, which facilitates Rad51 recruitment and repair by HR (Figure 8B). Although resection became faster, histone eviction was severely delayed in *bre1Δ* mutant. This was surprising because the *dot1Δ* mutant, which has a faster resection as seen in *bre1Δ* cells, displayed a faster kinetics in histone eviction than the WT cells. Thus, resection alone is not sufficient to drive histone disassembly, while uH2B appears to be indispensable for this process. Our data suggest that retention of histones on ssDNA impedes the loading of RPA and Rad51, slows down and impairs the repair by gene conversion. It is likely that loss of histones from ssDNA is necessary to provide space for the robust binding of RPA complex and Rad51 that spread over kilobases on ssDNA (11,12). In support of this notion, it has been reported that histones compete with HR proteins for binding damaged DNA (55). Consistently, we observed that overexpression of *RAD51* stimulates the histone eviction at DSBs in *bre1Δ* mutant and improves its HR efficiency and resistance to CPT (Figure 7A–D). Importantly, depletion of Cac1, a subunit of the CAF-1 histone chaperone that is involved in H3–H4 deposition during DNA replication or repair, significantly rescued the defect of *bre1Δ* mutant in HR repair (Supplementary Figure S14). We noted that DSB repair by SSA was not decreased in *bre1Δ* mutant, suggesting that uH2B is dispensable for the repair that does not need long Rad51 nucleofilaments. Thus, uH2B facilitates HR repair at least in part by regulating histone dynamics at DSBs.

Previous studies have shown that histones at DSB ends are partially evicted (11–13,16,17,60). In this study, we showed that the duplex DNA within 5 kb range at both sides of the DSB was completely or mostly resected after 4hr post break induction. However, we still detected considerable H3 occupancy within this region, suggesting that histones bind to ssDNA. Indeed, it has been shown that histone octamers can bind ssDNA efficiently and form nucleosome-like structures (56–58). It appears that the repair function of uH2B in HR is unrelated to H3K4 or H3K79 methylation and is independent of the ATP-dependent chromatin remodeling complexes Ino80, RSC, Fun30 and SWR (Supplementary Figure S11). These chromatin remodelers were known to regulate histone dynamics prior to resection (11–14,17). However, even in their presence, the lack of uH2B still led to delayed histone eviction, underscoring the indispensable role of uH2B in stimulating histone eviction and HR repair.

In human cells, it has been reported that uH2B-dependent H3K4 methylation at DNA breaks facilitates the recruitment of SNF2h, a subunit of ISW1 chromatin remodeling complex, which alters the local chromatin structure to allow the access of HR proteins and eventual repair by HR (42). Depletion of RNF20 or ablation of uH2B impairs resection and HR (41,42). In contrast, yeast cells lacking Bre1-uH2B exhibit a hyper resection phenotype. This difference could be due to that the recruitment of 53BP1 (the human homolog of yeast Rad9), which is known to inhibit resection, is dependent on H4K20 methylation and H2AK15 ubiquitination (61,62), two modifications independent of uH2B. While in yeast, the recruitment of Rad9 requires H3K79 methylation that is dependent on uH2B. Despite this difference, uH2B is important for the recruitment of repair proteins and repair by HR in both species, underscoring a conservative function of uH2B. However, it remains to be determined whether uH2B also regulates histone dynamics at DSBs in mammals.

There are several possibilities to explain how uH2B may act to regulate histone dynamics after resection. First, uH2B itself is able to disrupt chromatin compaction, creating an accessible chromatin context (18). This relaxed chromatin environment may facilitate histone eviction from ssDNA following resection. Second, uH2B may cooperate with the histone chaperone FACT as it does in transcription to displace H2A-H2B dimer thereby destabilizing nucleosomes given that FACT has also been linked to DNA repair (26). Finally, although uH2B acts independently of the ATP-dependent chromatin remodelers, we cannot rule out the possibility that it may facilitate the recruitment of other proteins that aid to regulate histone dynamics. Further studies are required to clarify these possibilities. Deregulation of uH2B machinery has been linked to multiple cancers (63,64). The E3 ligase RNF20 functions as a tumor suppressor and reduction in RNF20 levels causes defective HR and genome instability (63–65). Our findings provide novel insights into the regulation of HR by uH2B at additional layers, and aid to understand how the high levels of histones may down-regulate HR to preserve human genome integrity.

## DATA AVAILABILITY

The original RNA-seq data was deposited at GenBank under the SRA accession no. SRP131704.

## Supplementary Material

Supplementary Data

## References

[1] Bhattacharjee S., Nandi S. Choices have consequences: the nexus between DNA repair pathways and genomic instability in cancer. Clin. Transl. Med. 2016; 5:45. 27921283 10.1186/s40169-016-0128-z PMC5136664

[2] Symington L.S., Gautier J. Double-strand break end resection and repair pathway choice. Annu. Rev. Genet. 2011; 45:247–271. 21910633 10.1146/annurev-genet-110410-132435

[3] Hustedt N., Durocher D. The control of DNA repair by the cell cycle. Nat. Cell Biol. 2016; 19:1–9. 28008184 10.1038/ncb3452

[4] Zhao X., Wei C., Li J., Xing P., Li J., Zheng S., Chen X. Cell cycle-dependent control of homologous recombination. Acta Biochim. Biophys. Sin. (Shanghai). 2017; 49:655–668. 28541389 10.1093/abbs/gmx055

[5] Clerici M., Mantiero D., Lucchini G., Longhese M.P. The Saccharomyces cerevisiae Sae2 protein promotes resection and bridging of double strand break ends. J. Biol. Chem. 2005; 280:38631–38638. 16162495 10.1074/jbc.M508339200

[6] Garcia V., Phelps S.E., Gray S., Neale M.J. Bidirectional resection of DNA double-strand breaks by Mre11 and Exo1. Nature. 2011; 479:241–244. 22002605 10.1038/nature10515 PMC3214165

[7] Zhu Z., Chung W.H., Shim E.Y., Lee S.E., Ira G. Sgs1 helicase and two nucleases Dna2 and Exo1 resect DNA double-strand break ends. Cell. 2008; 134:981–994. 18805091 10.1016/j.cell.2008.08.037 PMC2662516

[8] Mimitou E.P., Symington L.S. Sae2, Exo1 and Sgs1 collaborate in DNA double-strand break processing. Nature. 2008; 455:U770–U773. 10.1038/nature07312 PMC3818707 18806779

[9] Bell J.C., Plank J.L., Dombrowski C.C., Kowalczykowski S.C. Direct imaging of RecA nucleation and growth on single molecules of SSB-coated ssDNA. Nature. 2012; 491:274–278. 23103864 10.1038/nature11598 PMC4112059

[10] Lisby M., Rothstein R. Cell biology of mitotic recombination. Cold Spring Harbor Perspect. Biol. 2015; 7:a016535. 10.1101/cshperspect.a016535 PMC4355273 25731763

[11] Chen X., Cui D., Papusha A., Zhang X., Chu C.D., Tang J., Chen K., Pan X., Ira G. The Fun30 nucleosome remodeller promotes resection of DNA double-strand break ends. Nature. 2012; 489:576–580. 22960743 10.1038/nature11355 PMC3640768

[12] Costelloe T., Louge R., Tomimatsu N., Mukherjee B., Martini E., Khadaroo B., Dubois K., Wiegant W.W., Thierry A., Burma S. The yeast Fun30 and human SMARCAD1 chromatin remodellers promote DNA end resection. Nature. 2012; 489:581–584. 22960744 10.1038/nature11353 PMC3493121

[13] Tsukuda T., Fleming A.B., Nickoloff J.A., Osley M.A. Chromatin remodelling at a DNA double-strand break site in Saccharomyces cerevisiae. Nature. 2005; 438:379–383. 16292314 10.1038/nature04148 PMC1388271

[14] van Attikum H., Fritsch O., Gasser S.M. Distinct roles for SWR1 and INO80 chromatin remodeling complexes at chromosomal double-strand breaks. EMBO J. 2007; 26:4113–4125. 17762868 10.1038/sj.emboj.7601835 PMC2230671

[15] Chen C.C., Carson J.J., Feser J., Tamburini B., Zabaronick S., Linger J., Tyler J.K. Acetylated lysine 56 on histone H3 drives chromatin assembly after repair and signals for the completion of repair. Cell. 2008; 134:231–243. 18662539 10.1016/j.cell.2008.06.035 PMC2610811

[16] Huang T.H., Fowler F., Chen C.C., Shen Z.J., Sleckman B., Tyler J.K. The histone chaperones ASF1 and CAF-1 promote MMS22L-TONSL-Mediated Rad51 loading onto ssDNA during homologous recombination in human cells. Mol. Cell. 2018; 69:879–892. 29478807 10.1016/j.molcel.2018.01.031 PMC5843376

[17] Shim E.Y., Hong S.J., Oum J.H., Yanez Y., Zhang Y., Lee S.E. RSC mobilizes nucleosomes to improve accessibility of repair machinery to the damaged chromatin. Mol. Cell. Biol. 2007; 27:1602–1613. 17178837 10.1128/MCB.01956-06 PMC1820475

[18] Fierz B., Chatterjee C., McGinty R.K., Bar-Dagan M., Raleigh D.P., Muir T.W. Histone H2B ubiquitylation disrupts local and higher-order chromatin compaction. Nat. Chem. Biol. 2011; 7:113–119. 21196936 10.1038/nchembio.501 PMC3078768

[19] Hwang W.W., Venkatasubrahmanyam S., Ianculescu A.G., Tong A., Boone C., Madhani H.D. A conserved RING finger protein required for histone H2B monoubiquitination and cell size control. Mol. Cell. 2003; 11:261–266. 12535538 10.1016/s1097-2765(02)00826-2

[20] Robzyk K., Recht J., Osley M.A. Rad6-dependent ubiquitination of histone H2B in yeast. Science. 2000; 287:501–504. 10642555 10.1126/science.287.5452.501

[21] Wood A., Krogan N.J., Dover J., Schneider J., Heidt J., Boateng M.A., Dean K., Golshani A., Zhang Y., Greenblatt J.F. Bre1, an E3 ubiquitin ligase required for recruitment and substrate selection of Rad6 at a promoter. Mol. Cell. 2003; 11:267–274. 12535539 10.1016/s1097-2765(02)00802-x

[22] Zhu B., Zheng Y., Pham A.D., Mandal S.S., Erdjument-Bromage H., Tempst P., Reinberg D. Monoubiquitination of human histone H2B: the factors involved and their roles in HOX gene regulation. Mol. Cell. 2005; 20:601–611. 16307923 10.1016/j.molcel.2005.09.025

[23] Dover J., Schneider J., Tawiah-Boateng M.A., Wood A., Dean K., Johnston M., Shilatifard A. Methylation of histone H3 by COMPASS requires ubiquitination of histone H2B by Rad6. J. Biol. Chem. 2002; 277:28368–28371. 12070136 10.1074/jbc.C200348200

[24] Ng H.H., Xu R.M., Zhang Y., Struhl K. Ubiquitination of histone H2B by Rad6 is required for efficient Dot1-mediated methylation of histone H3 lysine 79. J. Biol. Chem. 2002; 277:34655–34657. 12167634 10.1074/jbc.C200433200

[25] Sun Z.W., Allis C.D. Ubiquitination of histone H2B regulates H3 methylation and gene silencing in yeast. Nature. 2002; 418:104–108. 12077605 10.1038/nature00883

[26] Belotserkovskaya R., Oh S., Bondarenko V.A., Orphanides G., Studitsky V.M., Reinberg D. FACT facilitates transcription-dependent nucleosome alteration. Science. 2003; 301:1090–1093. 12934006 10.1126/science.1085703

[27] Ng H.H., Dole S., Struhl K. The Rtf1 component of the Paf1 transcriptional elongation complex is required for ubiquitination of histone H2B. J. Biol. Chem. 2003; 278:33625–33628. 12876293 10.1074/jbc.C300270200

[28] Van Oss S.B., Shirra M.K., Bataille A.R., Wier A.D., Yen K., Vinayachandran V., Byeon I.L., Cucinotta C.E., Heroux A., Jeon J. The histone modification domain of Paf1 complex subunit Rtf1 directly stimulates H2B Ubiquitylation through an interaction with Rad6. Mol. Cell. 2016; 64:815–825. 27840029 10.1016/j.molcel.2016.10.008 PMC5131541

[29] Wood A., Schneider J., Dover J., Johnston M., Shilatifard A. The Paf1 complex is essential for histone monoubiquitination by the Rad6-Bre1 complex, which signals for histone methylation by COMPASS and Dot1p. J. Biol. Chem. 2003; 278:34739–34742. 12876294 10.1074/jbc.C300269200

[30] Chandrasekharan M.B., Huang F., Sun Z.W. Ubiquitination of histone H2B regulates chromatin dynamics by enhancing nucleosome stability. PNAS. 2009; 106:16686–16691. 19805358 10.1073/pnas.0907862106 PMC2757834

[31] Fleming A.B., Kao C.F., Hillyer C., Pikaart M., Osley M.A. H2B ubiquitylation plays a role in nucleosome dynamics during transcription elongation. Mol. Cell. 2008; 31:57–66. 18614047 10.1016/j.molcel.2008.04.025

[32] Pavri R., Zhu B., Li G., Trojer P., Mandal S., Shilatifard A., Reinberg D. Histone H2B monoubiquitination functions cooperatively with FACT to regulate elongation by RNA polymerase II. Cell. 2006; 125:703–717. 16713563 10.1016/j.cell.2006.04.029

[33] Trujillo K.M., Osley M.A. A role for H2B ubiquitylation in DNA replication. Mol. Cell. 2012; 48:734–746. 23103252 10.1016/j.molcel.2012.09.019 PMC3525772

[34] Ma M.K., Heath C., Hair A., West A.G. Histone crosstalk directed by H2B ubiquitination is required for chromatin boundary integrity. PLos Genet. 2011; 7:e1002175. 21811414 10.1371/journal.pgen.1002175 PMC3140996

[35] Sadeghi L., Siggens L., Svensson J.P., Ekwall K. Centromeric histone H2B monoubiquitination promotes noncoding transcription and chromatin integrity. Nat. Struct. Mol. Biol. 2014; 21:236–243. 24531659 10.1038/nsmb.2776

[36] Wu Z., Liu J., Zhang Q.D., Lv D.K., Wu N.F., Zhou J.Q. Rad6-Bre1-mediated H2B ubiquitination regulates telomere replication by promoting telomere-end resection. Nucleic Acids Res. 2017; 45:3308–3322. 28180293 10.1093/nar/gkx101 PMC5389628

[37] Yamashita K., Shinohara M., Shinohara A. Rad6-Bre1-mediated histone H2B ubiquitylation modulates the formation of double-strand breaks during meiosis. PNAS. 2004; 101:11380–11385. 15280549 10.1073/pnas.0400078101 PMC509210

[38] Zofall M., Grewal S.I. HULC, a histone H2B ubiquitinating complex, modulates heterochromatin independent of histone methylation in fission yeast. J. Biol. Chem. 2007; 282:14065–14072. 17363370 10.1074/jbc.M700292200

[39] Chernikova S.B., Dorth J.A., Razorenova O.V., Game J.C., Brown J.M. Deficiency in Bre1 impairs homologous recombination repair and cell cycle checkpoint response to radiation damage in mammalian cells. Radiat. Res. 2010; 174:558–565. 20738173 10.1667/RR2184.1 PMC2988074

[40] Chernikova S.B., Razorenova O.V., Higgins J.P., Sishc B.J., Nicolau M., Dorth J.A., Chernikova D.A., Kwok S., Brooks J.D., Bailey S.M. Deficiency in mammalian histone H2B ubiquitin ligase Bre1 (Rnf20/Rnf40) leads to replication stress and chromosomal instability. Cancer Res. 2012; 72:2111–2119. 22354749 10.1158/0008-5472.CAN-11-2209 PMC3328627

[41] Moyal L., Lerenthal Y., Gana-Weisz M., Mass G., So S., Wang S.Y., Eppink B., Chung Y.M., Shalev G., Shema E. Requirement of ATM-dependent monoubiquitylation of histone H2B for timely repair of DNA double-strand breaks. Mol. Cell. 2011; 41:529–542. 21362549 10.1016/j.molcel.2011.02.015 PMC3397146

[42] Nakamura K., Kato A., Kobayashi J., Yanagihara H., Sakamoto S., Oliveira D.V., Shimada M., Tauchi H., Suzuki H., Tashiro S. Regulation of homologous recombination by RNF20-dependent H2B ubiquitination. Mol. Cell. 2011; 41:515–528. 21362548 10.1016/j.molcel.2011.02.002

[43] Zeng M., Ren L., Mizuno K., Nestoras K., Wang H., Tang Z., Guo L., Kong D., Hu Q., He Q. CRL4 (Wdr70) regulates H2B monoubiquitination and facilitates Exo1-dependent resection. Nat. Commun. 2016; 7:11364. 27098497 10.1038/ncomms11364 PMC4844679

[44] Game J.C., Chernikova S.B. The role of RAD6 in recombinational repair, checkpoints and meiosis via histone modification. DNA Repair (Amst.). 2009; 8:470–482. 19230796 10.1016/j.dnarep.2009.01.007

[45] Game J.C., Williamson M.S., Spicakova T., Brown J.M. The RAD6/BRE1 histone modification pathway in Saccharomyces confers radiation resistance through a RAD51-dependent process that is independent of RAD18. Genetics. 2006; 173:1951–1968. 16783014 10.1534/genetics.106.057794 PMC1569736

[46] Faucher D., Wellinger R.J. Methylated H3K4, a transcription-associated histone modification, is involved in the DNA damage response pathway. PLos Genet. 2010; 6:e1001082. 20865123 10.1371/journal.pgen.1001082 PMC2928815

[47] Ira G., Malkova A., Liberi G., Foiani M., Haber J.E. Srs2 and Sgs1-Top3 suppress crossovers during double-strand break repair in yeast. Cell. 2003; 115:401–411. 14622595 10.1016/s0092-8674(03)00886-9 PMC4493758

[48] Prakash R., Satory D., Dray E., Papusha A., Scheller J., Kramer W., Krejci L., Klein H., Haber J.E., Sung P. Yeast Mph1 helicase dissociates Rad51-made D-loops: implications for crossover control in mitotic recombination. Genes Dev. 2009; 23:67–79. 19136626 10.1101/gad.1737809 PMC2632165

[49] Vaze M.B., Pellicioli A., Lee S.E., Ira G., Liberi G., Arbel-Eden A., Foiani M., Haber J.E. Recovery from checkpoint-mediated arrest after repair of a double-strand break requires Srs2 helicase. Mol. Cell. 2002; 10:373–385. 12191482 10.1016/s1097-2765(02)00593-2

[50] Turco E., Gallego L.D., Schneider M., Kohler A. Monoubiquitination of histone H2B is intrinsic to the Bre1 RING domain-Rad6 interaction and augmented by a second Rad6-binding site on Bre1. J. Biol. Chem. 2015; 290:5298–5310. 25548288 10.1074/jbc.M114.626788 PMC4342449

[51] Briggs S.D., Xiao T., Sun Z.W., Caldwell J.A., Shabanowitz J., Hunt D.F., Allis C.D., Strahl B.D. Gene silencing: trans-histone regulatory pathway in chromatin. Nature. 2002; 418:498. 12152067 10.1038/nature00970

[52] Shahbazian M.D., Zhang K., Grunstein M. Histone H2B ubiquitylation controls processive methylation but not monomethylation by Dot1 and Set1. Mol. Cell. 2005; 19:271–277. 16039595 10.1016/j.molcel.2005.06.010

[53] Lazzaro F., Sapountzi V., Granata M., Pellicioli A., Vaze M., Haber J.E., Plevani P., Lydall D., Muzi-Falconi M. Histone methyltransferase Dot1 and Rad9 inhibit single-stranded DNA accumulation at DSBs and uncapped telomeres. EMBO J. 2008; 27:1502–1512. 18418382 10.1038/emboj.2008.81 PMC2328446

[54] Eapen V.V., Sugawara N., Tsabar M., Wu W.H., Haber J.E. The Saccharomyces cerevisiae chromatin remodeler Fun30 regulates DNA end resection and checkpoint deactivation. Mol. Cell. Biol. 2012; 32:4727–4740. 23007155 10.1128/MCB.00566-12 PMC3486187

[55] Liang D., Burkhart S.L., Singh R.K., Kabbaj M.H., Gunjan A. Histone dosage regulates DNA damage sensitivity in a checkpoint-independent manner by the homologous recombination pathway. Nucleic Acids Res. 2012; 40:9604–9620. 22850743 10.1093/nar/gks722 PMC3479188

[56] Adkins N.L., Swygert S.G., Kaur P., Niu H., Grigoryev S.A., Sung P., Wang H., Peterson C.L. Nucleosome-like, Single-stranded DNA (ssDNA)-Histone Octamer Complexes and the Implication for DNA Double Strand Break Repair. J. Biol. Chem. 2017; 292:5271–5281. 28202543 10.1074/jbc.M117.776369 PMC5392674

[57] Palter K.B., Foe V.E., Alberts B.M. Evidence for the formation of nucleosome-like histone complexes on single-stranded DNA. Cell. 1979; 18:451–467. 498278 10.1016/0092-8674(79)90064-3

[58] Wang Y., van Merwyk L., Tonsing K., Walhorn V., Anselmetti D., Fernandez-Busquets X. Biophysical characterization of the association of histones with single-stranded DNA. Biochim. Biophys. Acta. 2017; 1861:2739–2749. 10.1016/j.bbagen.2017.07.018 28756274

[59] Gurard-Levin Z.A., Quivy J.P., Almouzni G. Histone chaperones: assisting histone traffic and nucleosome dynamics. Annu. Rev. Biochem. 2014; 83:487–517. 24905786 10.1146/annurev-biochem-060713-035536

[60] Goldstein M., Derheimer F.A., Tait-Mulder J., Kastan M.B. Nucleolin mediates nucleosome disruption critical for DNA double-strand break repair. PNAS. 2013; 110:16874–16879. 24082117 10.1073/pnas.1306160110 PMC3801049

[61] Fradet-Turcotte A., Canny M.D., Escribano-Diaz C., Orthwein A., Leung C.C., Huang H., Landry M.C., Kitevski-LeBlanc J., Noordermeer S.M., Sicheri F. 53BP1 is a reader of the DNA-damage-induced H2A Lys 15 ubiquitin mark. Nature. 2013; 499:50–54. 23760478 10.1038/nature12318 PMC3955401

[62] Pei H., Zhang L., Luo K., Qin Y., Chesi M., Fei F., Bergsagel P.L., Wang L., You Z., Lou Z. MMSET regulates histone H4K20 methylation and 53BP1 accumulation at DNA damage sites. Nature. 2011; 470:124–128. 21293379 10.1038/nature09658 PMC3064261

[63] Shema E., Tirosh I., Aylon Y., Huang J., Ye C., Moskovits N., Raver-Shapira N., Minsky N., Pirngruber J., Tarcic G. The histone H2B-specific ubiquitin ligase RNF20/hBRE1 acts as a putative tumor suppressor through selective regulation of gene expression. Genes Dev. 2008; 22:2664–2676. 18832071 10.1101/gad.1703008 PMC2559905

[64] Wang E., Kawaoka S., Yu M., Shi J., Ni T., Yang W., Zhu J., Roeder R.G., Vakoc C.R. Histone H2B ubiquitin ligase RNF20 is required for MLL-rearranged leukemia. PNAS. 2013; 110:3901–3906. 23412334 10.1073/pnas.1301045110 PMC3593849

[65] Shema E., Kim J., Roeder R.G., Oren M. RNF20 inhibits TFIIS-facilitated transcriptional elongation to suppress pro-oncogenic gene expression. Mol. Cell. 2011; 42:477–488. 21596312 10.1016/j.molcel.2011.03.011 PMC3099049

