# Supplementary material for: Bre1-dependent H2B ubiquitination promotes homologous recombination by stimulating histone eviction at DNA breaks
Source: Nucleic Acids Res. 2018 Oct 10;46(21):11326–39. doi: 10.1093/nar/gky918 (PMC6265479; doi:10.1093/nar/gky918)
Supplement: Supplementary Data [file gky918_supplemental_files.zip › Supplementary Figures and legends.pdf]

Figure S1

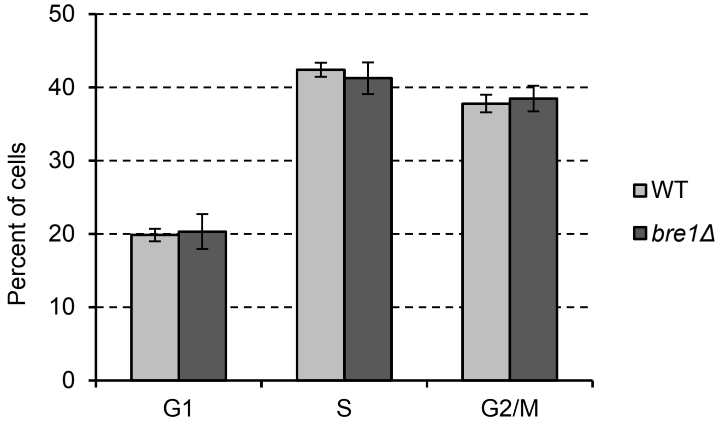

Figure S1. The absence of Bre1 does not alter cell cycle distribution. Asynchronized log phase WT and *bre1Δ* cells were collected and examined under microscope. Cell cycle phase was assessed based on bud size. At least 200 individual cells were counted for each strain in each experiment. Plotted values are average values ± SD from three independent experiments.

Figure S2

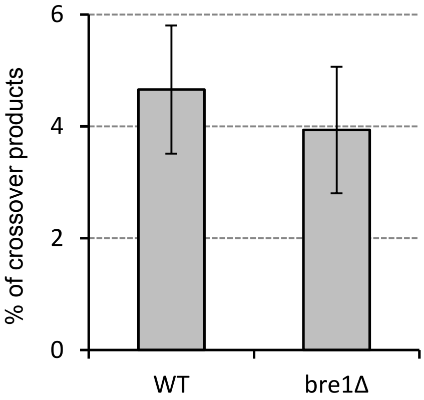

Figure S2. Crossover levels among repair products in WT and *bre1Δ* cells. Plotted values are average values ± SD from three independent experiments.

Figure S3

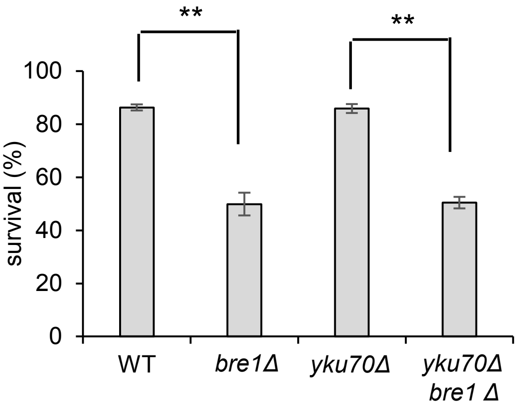

Figure S3. Survival rate for ectopic recombination in indicated strains. Plotted values are average values ± SD from three independent experiments. ** p<0.01(t-test)

Figure S4

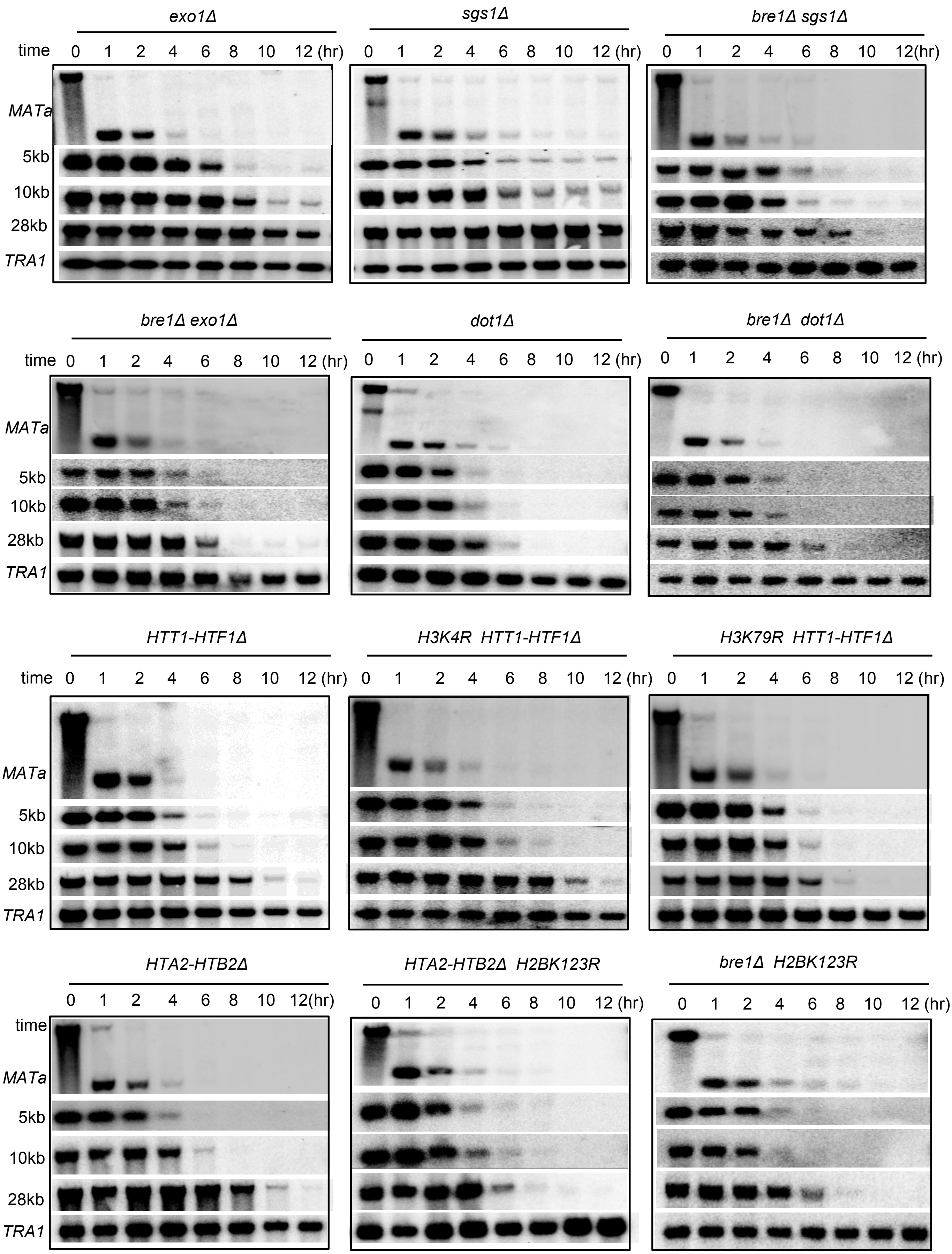
exo1Δ
sgs1Δ
bre1Δ sgs1Δ
time 0 1 2 4 6 8 10 12 (hr)
MATa
5kb
10kb
28kb
TRA1
bre1Δ exo1Δ
dot1Δ
bre1Δ dot1Δ
HTT1-HTF1Δ
H3K4R HTT1-HTF1Δ
H3K79R HTT1-HTF1Δ
HTA2-HTB2Δ
HTA2-HTB2Δ H2BK123R
bre1Δ H2BK123R

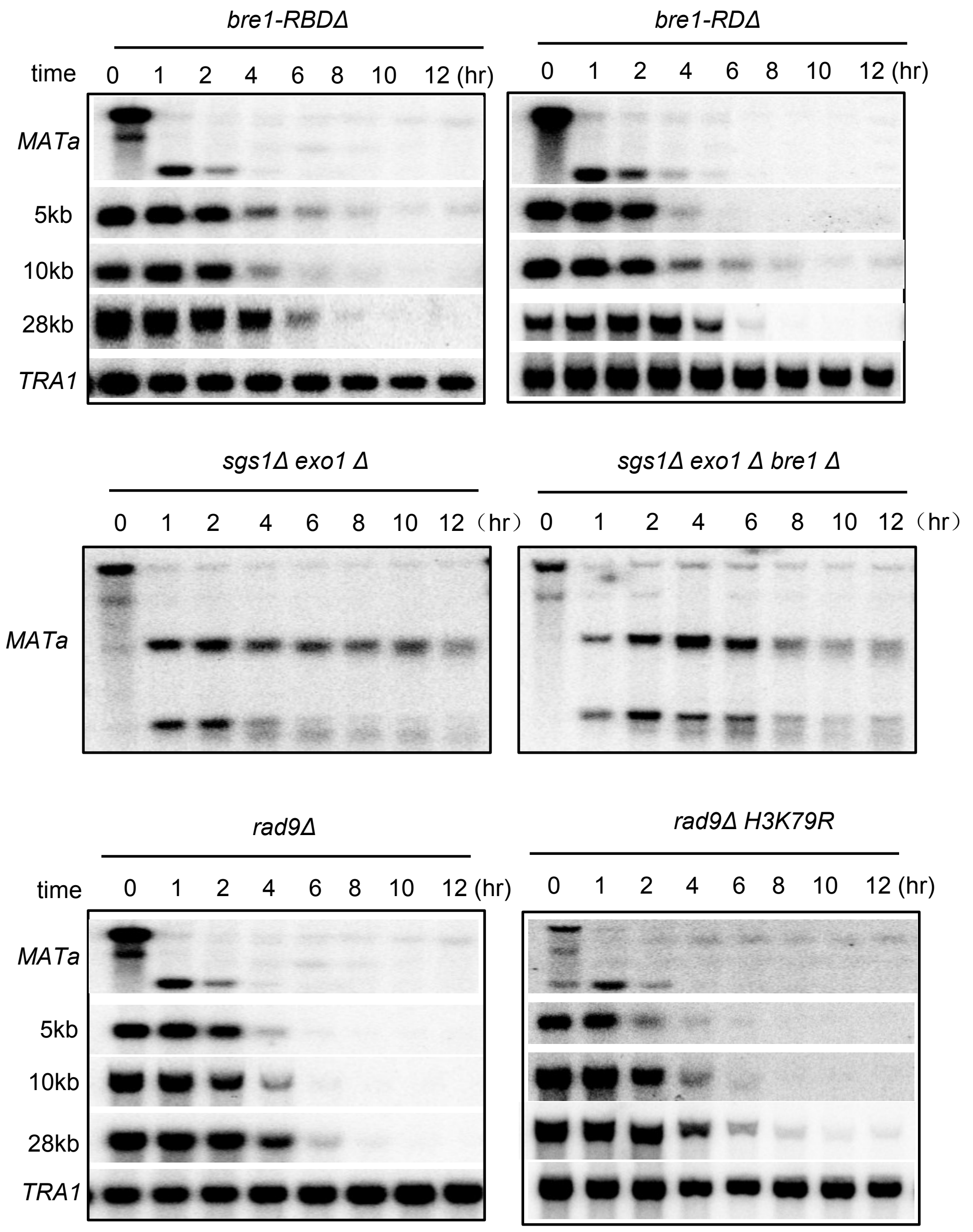

Figure S4. Southern blot analysis of resection kinetics for indicated strains. Cells were grown in YP-Raffinose to early log phase prior to break induction. Samples were collected at indicated time points following galactose Induction. DNA purification, restriction digestion and Southern blot were performed as described in Materials and Methods. The signal captured by a phosphor screen was detected using an OptiQuant Cyclone Plus machine (Perkin Elmer). *TRA1*, a locus on chromosome VIII where there are no DSBs, serves as a loading control.

Figure S5

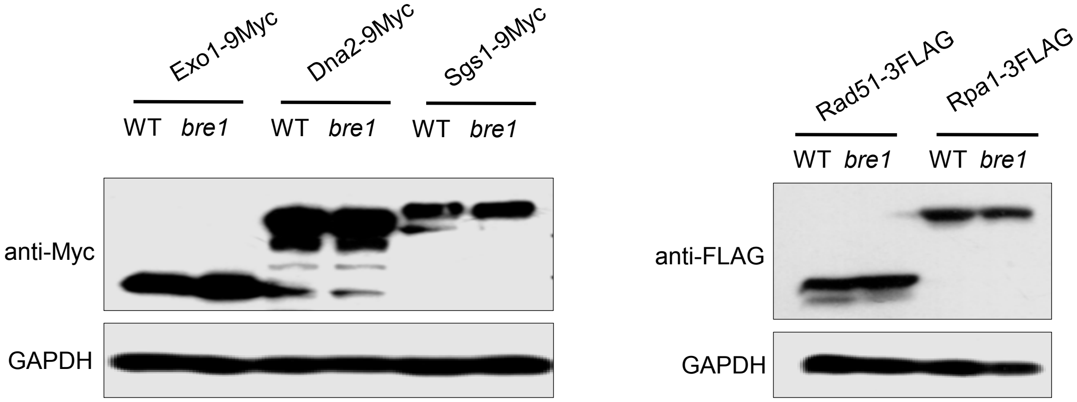

Figure S5. Western blot analysis of protein levels in the WT and *bre1Δ* mutant cells. Whole cell extracts were prepared using the TCA method. Anti-myc (Sigma) antibody was used to detect Exo1-9myc, Dna2-9myc and Sgs1-9myc proteins. Anti-FLAG (Sigma) antibodies were used to detect Rad51-3xFLAG and RPA-3xFLAG proteins. GAPDH represents a loading control.

Figure S6

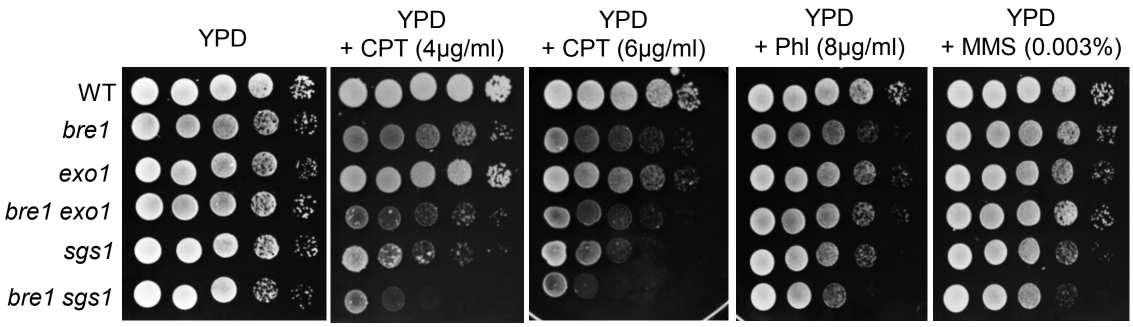

Figure S6. DNA damage sensitivity test for indicated strains. 10-fold dilutions of these strains on YPD plates without or with indicated DNA damaging agents. Plates were incubated at 30 ℃ for 2-3 days before taking picture.

Figure S7

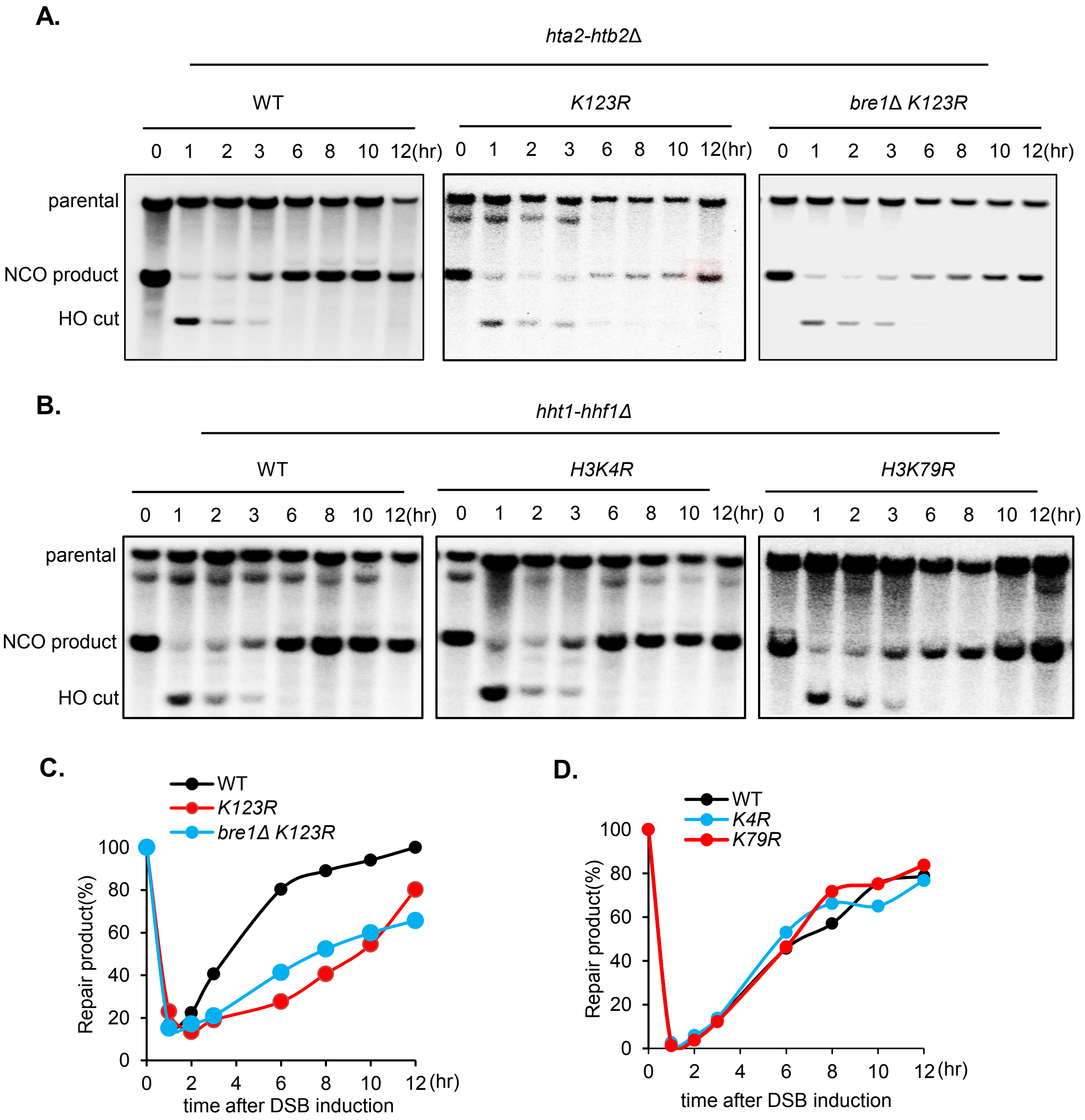

Figure S7. Southern blot analysis and quantification of ectopic recombination kinetics in indicated cells. A-B. Southern blot analysis of ectopic recombination kinetics in indicated strains. **C-D.** Quantification of the repair kinetics showed in A and B.

Figure S8

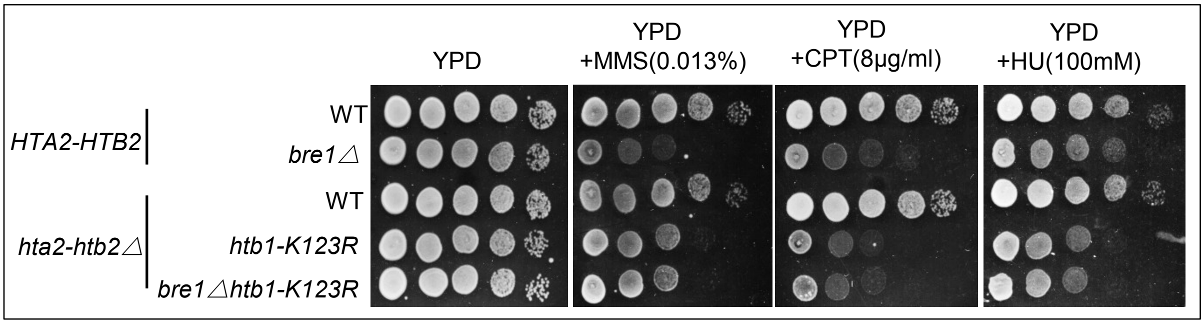

Figure S8. DNA damage sensitivity test for indicated strains. 10-fold dilutions of indicated strains on YPD plates without or with indicated DNA damaging agents**.** Plates were incubated at 30 ℃ for 2-3 days before taking picture.

Figure S9

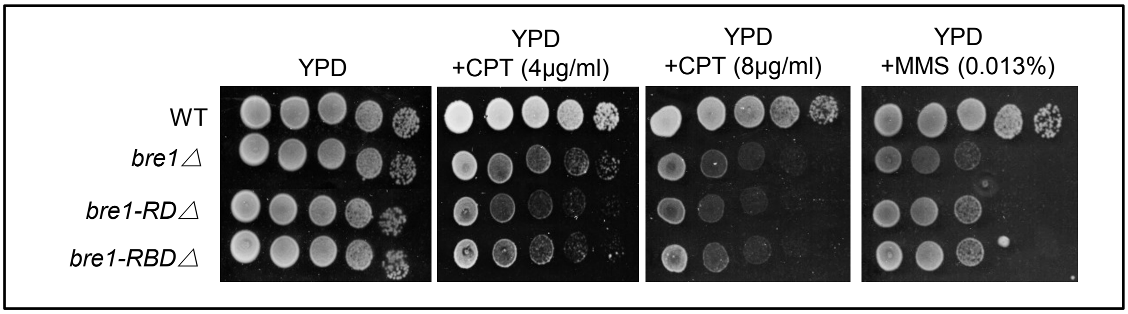

Figure S9. DNA damage sensitivity test for indicated strains. 10-fold dilutions of indicated strains on YPD plates with or without indicated DNA damaging-agents**.** Plates were incubated at 30 ℃ for 2-3 days before taking picture.

Figure S10

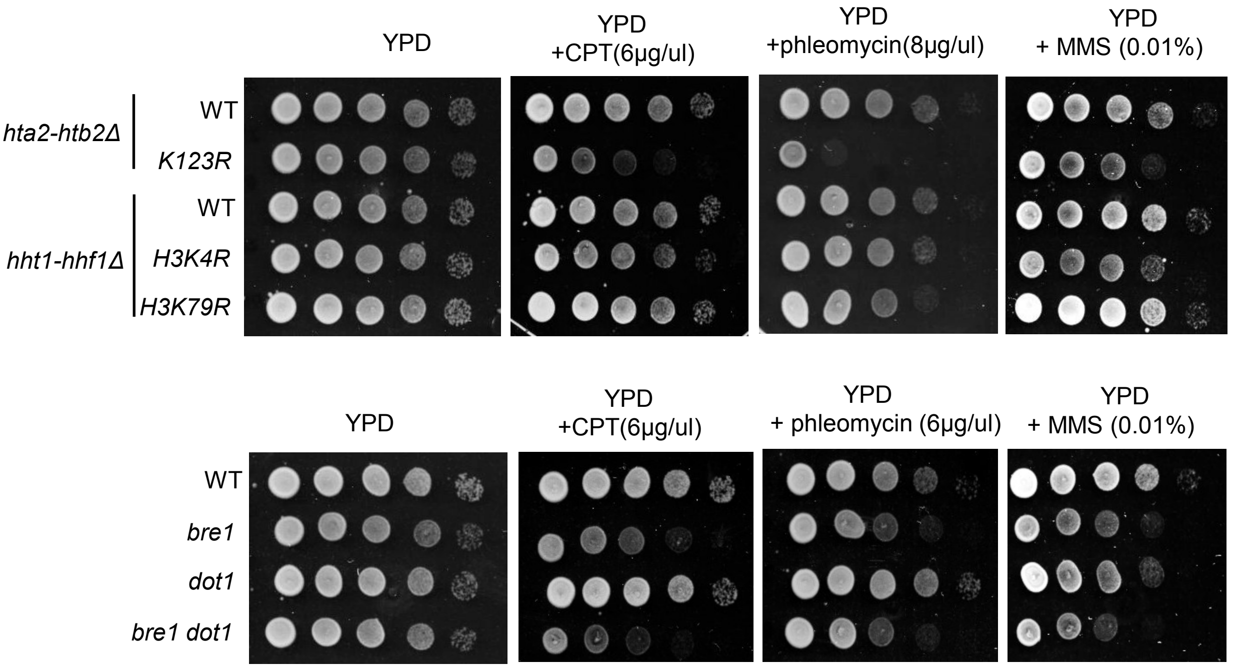

Figure S10. DNA damage sensitivity test for indicated strains. 10-fold dilutions of indicated strains on YPD plates with or without indicated DNA damaging-agents**.** Plates were incubated at 30 ℃ for 2-3 days before taking picture.

Figure S11

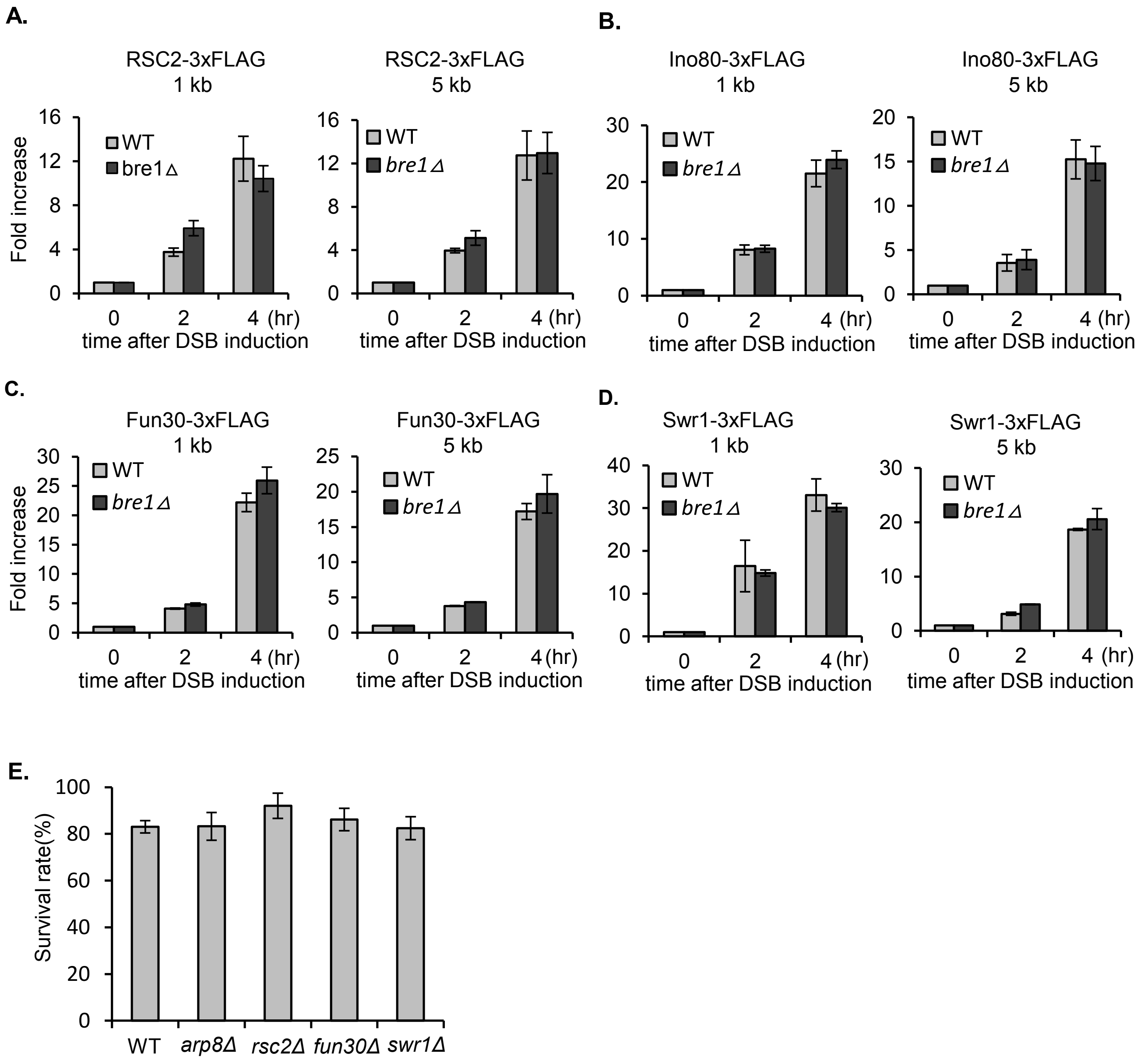

Figure S11. The role of uH2B in HR repair is independent of ATP-dependent chromatin remodelers. **A-D**. ChIP-qPCR analysis of the enrichment for Rsc2-3xFLAG, Ino80-3xFLAG, Fun30-3xFLAG and Swr1-3xFLAG at 1 or 5 kb location in WT or *bre1Δ* cells. **E.** Survival rate for repair by ectopic recombination. Plotted values are mean values ± SD from three independent experiments.

Figure S12

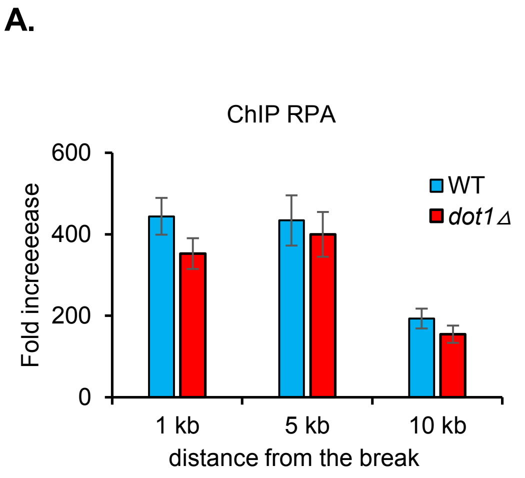

Figure S12. Deletion of *DOT1* does not impair the recruitment of RPA and Rad51. ChIP-qPCR analysis of RPA-3xFLAG and Rad51-3xFLAG recruitment at indicated locations 4hr following DSB induction in WT and *dot1Δ* cells. Error bar denotes standard deviation from three independent experiments.

Figure S13

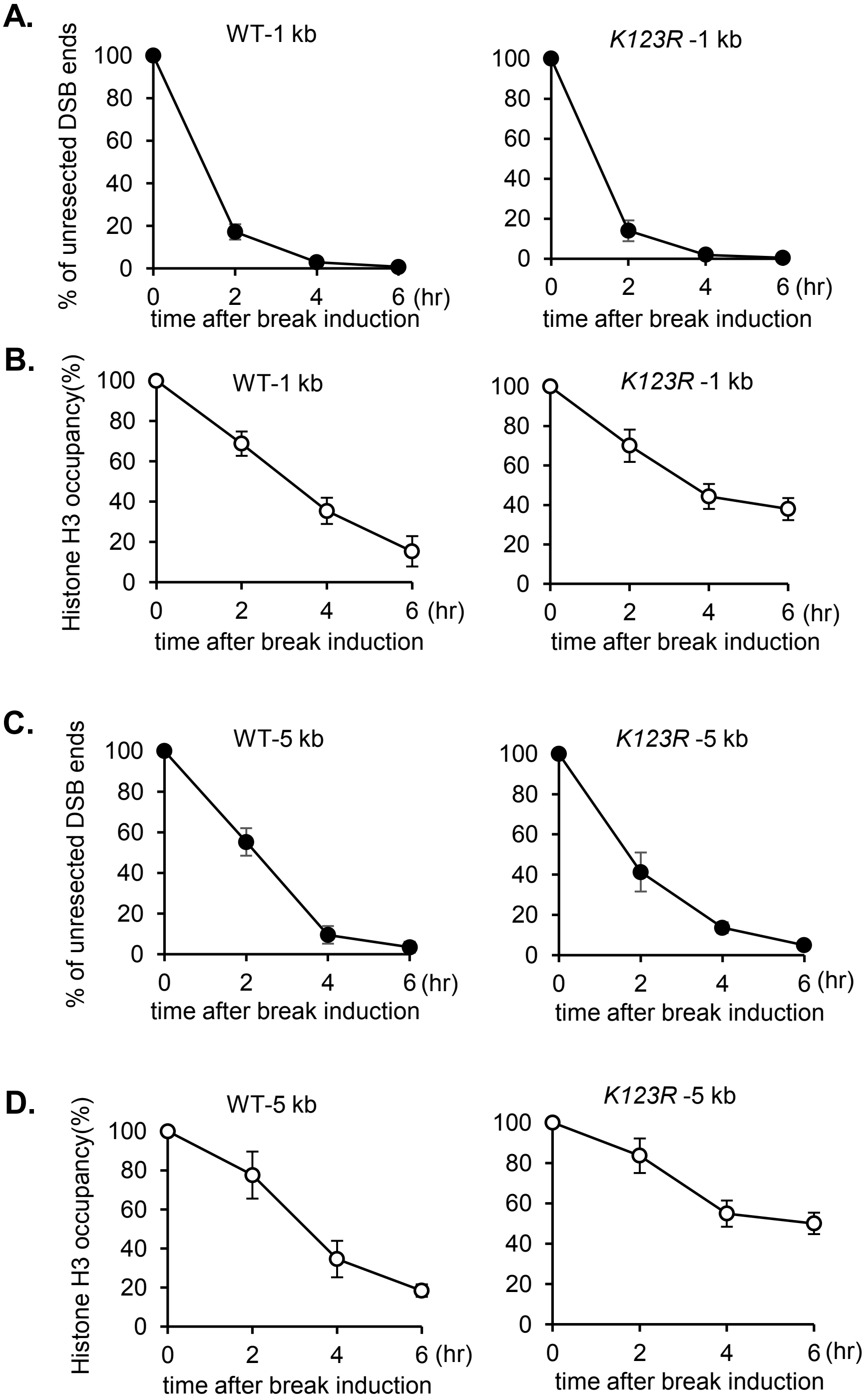

Figure S13. H2B ubiquitylation stimulates histone loss following resection. A and C. Quantification of resection kinetics at 1 or 5 kb location for WT cells and *K123R* mutant. The corresponding Southern blot is presented in Figure S4. **B and D.** ChIP-qPCR analysis of histone H3 occupancy at 1 or 5 kb location for WT cells and *K123R* mutant. Three independent experiments were performed for each strain. Plotted values are mean values ± SD from three independent experiments.

Figure S14

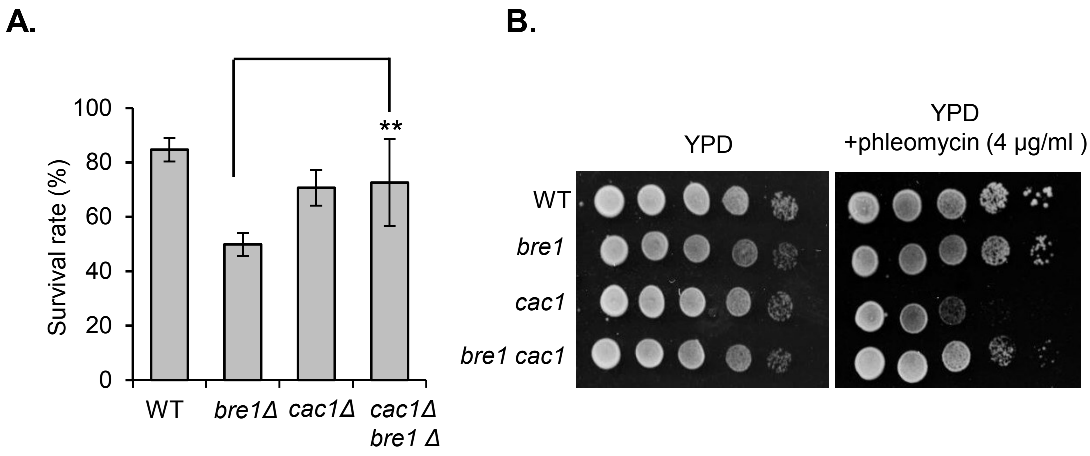

Figure S14. The genetic interaction between Bre1 and CAF-1. A. Survival rate for repair by ectopic recombination. Plotted values are mean values ± SD from three independent experiments. ** represents statistical significance (p<0.01, t-test). **B**. drug sensitivity test for indicated cells on phleomycin.
