# Supplementary material for: Bre1-dependent H2B ubiquitination promotes homologous recombination by stimulating histone eviction at DNA breaks
Source: Nucleic Acids Res. 2018 Oct 10;46(21):11326–39. doi: 10.1093/nar/gky918 (PMC6265479; doi:10.1093/nar/gky918)
Supplement: Supplementary Data [file gky918_supplemental_files.zip › Supplementary Table 1.pdf]

Supplementary Table 1. Yeast strains used in this study

Strain name	Parental strain	Genotype	Source
JKM139		*MATa ho hml::ADE1 hmr::ADE1 ade1-100 leu2-3,112 trp1::hisG lys5 ura3-52 ade3::GAL::HO*	1
JKM179		*MATalpha ho hml::ADE1 hmr::ADE1 ade1-100 leu2-3,112 trp1::hisG lys5 ura3-52 ade3::GAL::HO*	1
yCW05	JKM139	*Rad51-3xFLAG-KanMX*	This study
yDL022	JKM139	*Rad51-3xFLAG-KanMX bre1::TRP1*	This study
yDL070	JKM139	*Rad51-3xFLAG-KanMX dot1::URA3*	This study
yDL060	JKM139	*Rad51-3xFLAG-KanMX hta2-htb2::KanMX::URA3*	This study
yZSH270	JKM139	*Rad51-3xFLAG-KanMX hta2-htb2::LEU2 htb1-K123R-TRP1*	This study
yCW07	JKM139	*Rfa1-3xFLAG-KanMX*	This study
yDL023	JKM139	*Rfa1-3xFLAG-KanMX bre1::TRP1*	This study
yDL071	JKM139	*Rfa1-3xFLAG-KanMX dot1::URA3*	This study
yDL056	JKM139	*Rfa1-3xFLAG-KanMX hta2-htb2::LEU2 htb1-K123R-TRP1*	This study
yDL061	JKM139	*Rfa1-3xFLAG-KanMX hta2-htb2::KanMX::URA3*	This study
yZZ042	JKM139	*Exo1-9xMyc-TRP1*	2
yDL021	JKM139	*Dna2-9xMyc-TRP1 bre1:: KanMX*	This study
yXC002	JKM139	*Dna2-9xMyc-TRP1*	2
yDL026	JKM139	*Dna2-9xMyc-TRP1 bre1:: KanMX*	This study
yZSH167	JKM139	*Sgs1-9xMyc-TRP1*	2
yDL027	JKM139	*Sgs1-9xMyc-TRP1 bre1:: KanMX*	This study
yDL044	JKM139	*Bre1-3xFLAG-KanMX*	This study
yZSH280	JKM179	*Bre1-3xFLAG-KanMX h2a-S129A*	This study
yZSH281	JKM139	*Bre1-3xFLAG-KanMX sgs1::NatMX exo1::TRP1*	This study
yDL052	JKM139	*Bre1-3xFLAG-KanMX mec1::NatMX tel1::LEU2 sml1::TRP1*	This study
yCW35	JKM139	*yKu70-3xFLAG-KanMX*	This study
yDL040	JKM139	*yKu70-3xFLAG-KanMX bre1::TRP1*	This study
yDL065	JKM139	*yKu70-3xFLAG-KanMX hta2-htb2::LEU2 htb1-K123R-TRP1*	This study
yDL066	JKM139	*yKu70-3xFLAG-KanMX hta2-htb2::KanMX::URA3*	This study
yXC709	JKM179	*FLAG-HHT1::LEU2*	2
yZSH283	JKM139	*FLAG-HHT1::LEU2 bre1:: KanMX*	This study

yZSH286	JKM139	*FLAG-HHT1::LEU2 dot1:: KanMX*	This study
yDL030	JKM139	*bre1::KanMX*	This study
yZSH258	JKM139	*bre1-RBDΔ::TRP1*	This study
yZSH238	JKM139	*bre1-RDΔ::TRP1*	This study
yXC1047	JKM139	*yku70::NatMX cdc28-as1*	This study
yDL032	JKM139	*bre1:: KanMX yku70::NatMX cdc28-as1*	This study
yXC219	JKM139	*exo1::KanMX*	This study
yDL029	JKM139	*bre1::TRP1 exo1::KanMX*	This study
yXC548	JKM139	*sgs1::NatMX*	This study
yDL035	JKM139	*bre1::URA3 sgs1::NatMX*	This study
yZSH336	JKM139	*bre1::KanMX sgs1::NatMX exo1::TRP1*	This study
yXC780	JKM139	*dot1::KanMX*	2
yZL017	JKM139	*dot1::URA3 bre1::KanMX*	This study
yWM005	JKM139	*hta2-htb2::KanMX*	This study
yZSH246	JKM139	*hta2-htb2::KanMX htb1-K123R-TRP1*	This study
yZSH271	JKM139	*hta2-htb2::LEU2 htb1-K123R-TRP1 bre1::KanMX*	This study
yJL033	JKM139	*hht1-hhf1::URA3*	This study
yZSH202	JKM139	*hht1-hhf1::URA3 hht2-K4R-TRP1*	This study
yZL004	JKM139	*hht1-hhf1::URA3 hht2-K79R-TRP1*	This study
yZSH335	JKM139	*hht1-hhf1::URA3 hht2-K79R-TRP1 rad9::KanMX*	This study
ySY124	JKM139	*Rad52-YFP-KanMX*	This study
yZSH242	JKM139	*Rad52-YFP-KanMX bre1::KanMX*	This study
yZSH262	JKM139	*Rad52-YFP-KanMX hta2-htb2::Leu2*	This study
yZSH261	JKM139	*Rad52-YFP-KanMX hta2-htb2::Leu2 htb1-K123R-TRP1*	This study
yDL048	JKM139	*bre1:: KanMX + pRS316*	This study
yDL049	JKM139	*bre1:: KanMX + pRS316-BRE1*	This study
yZSH284	JKM139	*bre1::KanMX+pRS426-Rad51*	This study
yZSH297	JKM139	*bre1::KanMX FLAG-HHT1-LEU2 +pRS426*	This study
yZSH296	JKM139	*bre1::KanMX FLAG-HHT1-LEU2 +pRS426-Rad51*	This study
yZSH327	JKM139	*cac1::KanMX*	This study
yZSH328	JKM139	*cac1::KanMX bre1::URA3*	This study
tGI354		*MATa-inc arg5,6::MATa-HPH ade3::GAL::HO hmr::ADE1 hml::ADE1 ura3-52*	3
yDL025	tGI354	*bre1::KanMX*	This study
yZSH259	tGI354	*bre1-RBD Δ::TRP1*	This study
yZSH333	tGI354	*yku70::NatMX*	This study
yZSH334	tGI354	*yku70::NatMX bre1-RDΔ::TRP1*	This study
yZL033	tGI354	*hta2-htb2::KanMX htb1-K123R-TRP1*	This study
yZSH276	tGI354	*hta2-htb2::LEU2 htb1-K123R-TRP1 bre1::KanMX*	This study
yZL007	tGI354	*hht1-hhf1::TRP1*	This study
yZL020	tGI354	*hht1-hhf1::TRP1 hht2-K4R- URA3*	This study
yZL027	tGI354	*hht1-hhf1::TRP1 hht2-K79R- URA3*	This study

yZSH292	tGI354	*bre1::KanMX+pRS426*	This study
yZSH285	tGI354	*bre1::KanMX+pRS426-Rad51*	This study
yZSH329	tGI354	*cac1::KanMX*	This study
yZSH330	tGI354	*cac1::KanMX bre1::TRP1*	This study
yMV080		*ho hml::ADE1 mata::hisG hmr::ADE1 his4::NatMX-leu2 (XhoI to Asp718) leu2::MATa ade3::GAL::HO ade1 lys5 ura3- 52 trp1*	4
yWH378	yMV080	*rad51::URA3*	4
yDL033	yMV080	*rad51::URA3 bre1::KanMX*	This study

1. Lee, S. E. et al., Saccharomyces Ku70, mre11/rad50 and RPA proteins regulate adaptation to G2/M arrest after DNA damage.(1998). Cell 94 (3): 399.

2. Chen, X. et al., The Fun30 nucleosome remodeller promotes resection of DNA double-strand break ends. (2012). Nature 489(7417):576-80.

3. Ira G. et al., Srs2 and Sgs1-Top3 suppress crossovers during double-strand break repair in yeast. (2003). Cell.115(4):401-11.

4. Zhu, Z. et al., Sgs1 helicase and two nucleases dna2 and exo1 resect DNA doublestrand break ends.(2008) Cell 134 (6): 981
